# CD69 expression on regulatory T cells protects from immune damage after myocardial infarction

**DOI:** 10.1172/JCI152418

**Published:** 2022-11-01

**Authors:** Rafael Blanco-Domínguez, Hortensia de la Fuente, Cristina Rodríguez, Laura Martín-Aguado, Raquel Sánchez-Díaz, Rosa Jiménez-Alejandre, Iker Rodríguez-Arabaolaza, Andrea Curtabbi, Marcos M. García-Guimaraes, Alberto Vera, Fernando Rivero, Javier Cuesta, Luis J. Jiménez-Borreguero, Alberto Cecconi, Albert Duran-Cambra, Manel Taurón, Judith Alonso, Héctor Bueno, María Villalba-Orero, Jose Antonio Enríquez, Simon C. Robson, Fernando Alfonso, Francisco Sánchez-Madrid, José Martínez-González, Pilar Martín

**Affiliations:** 1 Vascular Pathophysiology Area, Centro Nacional de Investigaciones Cardiovasculares (CNIC), Madrid, Spain.; 2 Department of Immunology, IIS Princesa, Hospital Universitario de la Princesa, Universidad Autónoma de Madrid, Madrid, Spain.; 3 CIBER de Enfermedades Cardiovasculares (CIBERCV), Madrid, Spain.; 4 Institut de Recerca del Hospital de la Santa Creu i Sant Pau, Barcelona, Spain.; 5 Instituto de Investigación Biomédica Sant Pau (IIB-Sant Pau), Barcelona, Spain.; 6 Department of Cardiology, IIS Princesa, Hospital Universitario de la Princesa, Universidad Autónoma de Madrid, Madrid, Spain.; 7 Hospital de la Santa Creu i Sant Pau, Barcelona, Spain.; 8 Instituto de Investigaciones Biomédicas de Barcelona–Consejo Superior de Investigaciones Científicas (IIBB-CSIC), Barcelona, Spain.; 9 Cardiology Department, Hospital Universitario 12 de Octubre and Instituto de Investigación Sanitaria Hospital 12 de Octubre (imas12), Madrid, Spain.; 10 Facultad de Medicina, Universidad Complutense de Madrid, Madrid, Spain.; 11 Departamento de Medicina y Cirugía Animal, Facultad de Veterinaria, Universidad Complutense de Madrid, Madrid, Spain.; 12 CIBER de Fragilidad y Envejecimiento Saludable (CIBERFES), Madrid, Spain.; 13 Department of Medicine, Harvard Medical School, Transplantation Research Center, Beth Israel Deaconess Medical Center, Boston, Massachusetts, USA.

**Keywords:** Cardiology, Immunology, Cardiovascular disease, Heart failure, T cells

## Abstract

Increasing evidence has pointed to the important function of T cells in controlling immune homeostasis and pathogenesis after myocardial infarction (MI), although the underlying molecular mechanisms remain elusive. In this study, a broad analysis of immune markers in 283 patients revealed significant CD69 overexpression on Tregs after MI. Our results in mice showed that CD69 expression on Tregs increased survival after left anterior descending (LAD) coronary artery ligation. *Cd69^–/–^* mice developed strong IL-17^+^ γδT cell responses after ischemia that increased myocardial inflammation and, consequently, worsened cardiac function. CD69^+^ Tregs, by induction of AhR-dependent CD39 ectonucleotidase activity, induced apoptosis and decreased IL-17A production in γδT cells. Adoptive transfer of CD69^+^ Tregs into *Cd69^–/–^* mice after LAD ligation reduced IL-17^+^ γδT cell recruitment, thus increasing survival. Consistently, clinical data from 2 independent cohorts of patients indicated that increased CD69 expression in peripheral blood cells after acute MI was associated with a lower risk of rehospitalization for heart failure (HF) after 2.5 years of follow-up. This result remained significant after adjustment for age, sex, and traditional cardiac damage biomarkers. Our data highlight CD69 expression on Tregs as a potential prognostic factor and a therapeutic option to prevent HF after MI.

## Graphical abstract

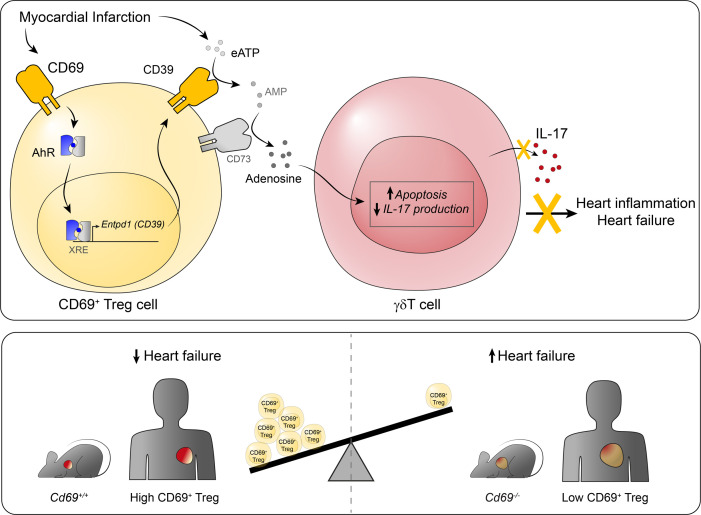

## Introduction

Myocardial infarction (MI) is an acute ischemic myocardial insult that clinically represents the global leading cause of mortality. Inflammation plays an essential role in the pathophysiology of atherosclerosis, plaque formation, and plaque rupture, the main trigger of MI, but also in the process of myocardial healing and repair after ischemia-induced damage (1). The role of innate immunity and myeloid cells after MI has been widely studied (1, 2), but that of the adaptive immunity remains scarcely explored. Recent studies have shown that CD4^+^ T cells are important contributors to inflammatory mechanisms and, more specifically, to myocardial damage repair mechanisms after ischemia-reperfusion processes (3, 4). Indeed, CD4^+^CD25^+^Foxp3^+^ Tregs have emerged as important players in protection against cardiac damage after experimental MI (5). MI induces a cardioprotective T cell response (6) and a rapid recruitment of Tregs to the injured myocardium, mediated by the CXCL12/CXCR4 axis (7) and galectin-1 (Gal-1) (8). In parallel, Tregs become activated 7 days after MI in the heart-draining lymph nodes (9) with increased percentages and absolute numbers, protecting against uncontrolled inflammation and cardiac deterioration. Indeed, Treg depletion aggravates cardiac inflammation and worsens prognosis early after experimental MI (9, 10). However, the specific molecular mechanisms that operate in these processes remain elusive.

Different studies in patients indicate that the proportions of Tregs change in peripheral blood after MI (11–13). In addition, some evidence indicates that Tregs accumulate in coronary thrombi (14) and in the heart (6) of patients with MI, reinforcing the notion that Tregs play a major role at the site of injury. Studies in larger cohorts of patients and exploration of different Treg regulatory molecules may further clarify the function of these cells after MI.

CD69 is a C-type lectin whose gene is encoded within the NK cell gene complex on chromosome 6 in mice and chromosome 12 in humans. CD69 promotes immune homeostasis by regulating the differentiation and function of Tregs (15, 16). On the basis of CD69 expression, Tregs are classified into 2 subtypes: CD69^+^ and CD69^–^ Tregs. CD69^+^ Tregs express higher levels of suppression markers such as CTLA-4, ICOS, CD38, and GITR than to CD69^–^ or *Cd69^–/–^* Tregs, resulting in enhanced effector T cell suppression and tolerance (15). *Cd69^–/–^* mice exhibit uncontrolled Th17 cell (CD3^+^CD4^+^IL-17^+^) responses and aggravation of multiple autoimmune and inflammatory disease models (17), including those of the cardiovascular system such as atherosclerosis (18), myocarditis, and inflammatory dilated cardiomyopathy (19). Other studies highlight that IL-17 exacerbates fibrosis and cardiac remodeling after MI (20, 21), although the involvement of Th17 cells after cardiac injury remains controversial. CD3^+^TCRγδ^+^ (γδT) cells, which rapidly accumulate in the infarcted myocardium (22), are the main source of IL-17 during the first week after MI and appear to be associated with the morbidity of MI in mice (23) and patients (24).

There is evidence linking CD69 expression on T cells to MI. Interestingly, natural ligands of CD69, such as Gal-1 (25) or S100A8/A9 (26), are upregulated after MI and contribute to immune cell recruitment (8, 27). Furthermore, CD69 is overexpressed on infiltrating T cells in the atherosclerotic plaques (28) and circulating CD4^+^ T lymphocytes of patients with MI, in whom it has been associated with increased apoptosis driven by lymphocyte activation (29). Therefore, we postulated that CD69 expression on lymphocytes might be a player in the post-MI progression toward chronic scenarios.

Many classical clinical and anatomic factors have been involved in the prognosis of patients with MI. In these patients, early reperfusion followed by guideline-based medical therapy represents the cornerstone of clinical management. However, predictive models, capable of identifying MI patients at higher risk for adverse long-term clinical outcomes, remain limited (30). Consequently, the use of additional markers capable of improving risk stratification after MI to select patients most likely to benefit from closer clinical follow-up, or more aggressive therapeutic strategies, would be desirable.

This study reveals that increased expression of CD69^+^ Tregs is a common feature of mouse and patient peripheral blood shortly after MI. In addition, we describe a mechanism by which CD69^+^ Tregs controlled inflammation and cardiac damage after coronary artery ligation through inhibition of pathogenic IL-17^+^ γδT cells. Finally, in the clinical setting, we found that Tregs with low CD69 expression after acute MI were associated with an increased risk of rehospitalization for heart failure (HF) at mid-term follow-up. Therefore, our data suggest that increasing the expression of CD69 on Tregs could be a promising strategy to improve post-MI management of patients.

## Results

### Enhanced peripheral CD69^+^ Treg response in patients with acute MI.

**Figure 1:**
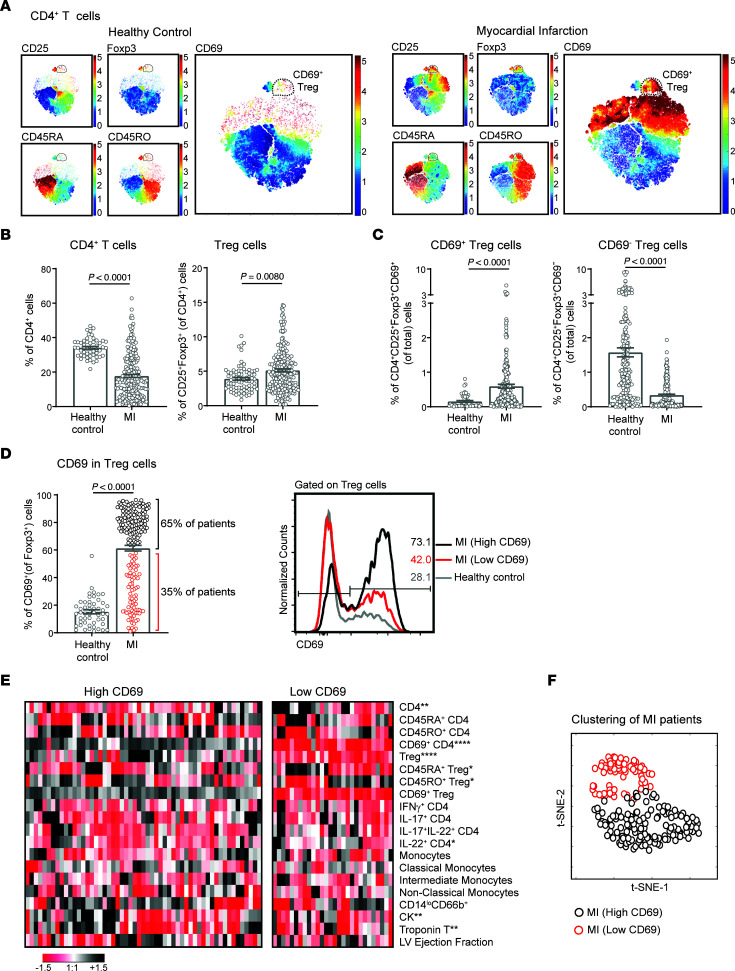
Patients with MI have a strong peripheral CD69^+^ Treg response. (**A**) *t*-SNE plots of CD4^+^ T cells from PBLs from a representative healthy control and a patient with MI, considering the indicated markers measured by FACS. Color bars indicate the relative intensity of the markers. Dots represent individual cells. (**B**) Quantification of the percentages of CD4^+^ T cells and CD4^+^CD25^+^Foxp3^+^ Tregs in peripheral blood from healthy donors (*n* = 51) and patients with MI (*n* = 215) at the time of hospital admission. (**C**) Percentages of CD69^+^ Tregs and CD69^–^ Tregs among total PBLs. (**D**) CD69 expression on Tregs, quantified as the percentage of CD69^+^ cells after gating on Tregs. Two groups of patients with MI were differentiated according to CD69 expression: patients with high levels of CD69 (High CD69), shown as black circles, and patients with low levels (Low CD69), shown as red circles. Representative histograms and percentages of CD69 expression on Tregs are shown. In **B**–**D**, data indicate the mean ± SEM, and significance was analyzed by Mann-Whitney *U* test. (**E**) Heatmap shows the levels of different cell populations analyzed by FACS and cardiac damage markers in patient with MI expressing high levels of CD69 and low levels of CD69. Each column represents 1 patient. Data were normalized by subtracting the mean and dividing by the SD. Color bar denotes the relative levels of each parameter, with black indicating high expression and red indicating low expression. Differences between patients with CD69^hi^ and CD69^lo^ expression were analyzed by Mann-Whitney *U* test; *P < 0.05, **P < 0.01, and ****P < 0.0001. (**F**) *t*-SNE plot was generated based on the percentages of cell populations shown in **E** and analyzed by FACS. Circles represent individual patients with MI. Black circles indicate patients with MI who had high CD69 expression, and red circles indicate patients with low CD69 expression.

The blood immune phenotype was determined in a cohort of 283 participants with acute MI and 80 healthy volunteers (Figure 1). Demographic and clinical data are summarized in Supplemental Table 1; supplemental material available online with this article; https://doi.org/10.1172/JCI152418DS1 More than 90% of patients presented with at least 1 risk factor, most frequently dyslipidemia, arterial hypertension, and a history of smoking. Supplemental Table 1 also summarizes the results of biomarkers, ECG, coronary angiography (number of diseased vessels), and echocardiography (left ventricular [LV] segmental wall motion abnormalities and ejection fraction). Most patients presented with ST-segment elevation MI, and the culprit lesion was more often located in the left anterior descending (LAD) coronary artery or the right coronary artery, with 38.5% of patients having multivessel coronary artery disease. LV systolic function was generally preserved, and only one-third of patients had a reduced LV ejection fraction.

We performed an extensive analysis of lymphoid and myeloid cell populations by FACS in peripheral blood in the first 24 hours after the ischemic event (Supplemental Figure 1A). Elevation of serum cardiac damage markers, such as troponin T (TnT) and creatine kinase (CK), after MI were correlated with CD69 expression on circulating CD4^+^ T cells and CD4^+^CD25^+^Foxp3^+^ Tregs as well as with the ratios of effector T cells versus CD69^+^ Tregs, suggesting that CD69 expression was induced upon myocardial damage (Supplemental Table 2 and Supplemental Figure 1A). Indeed, the analysis of CD4^+^ T cells by flow cytometry revealed that a subset of CD69^+^CD25^+^Foxp3^+^ Tregs appeared after MI (Figure 1A). CD4^+^ T cell and Treg populations were decreased and augmented, respectively, in patients with MI compared with healthy controls (Figure 1B and Supplemental Figure 1B). CD69 is virtually absent in circulating blood lymphocytes due to the high concentration of sphingosine-1-phosphate (S1P), which maintains S1P_1_ receptors at high levels (31), thereby suppressing CD69 expression (32). Therefore, we analyzed CD69 expression in circulating CD4^+^ T cells in the blood after overnight bystander activation with anti-CD3 antibody. The percentage of CD69^+^ Tregs increased in the circulation in patients with MI (Figure 1C and Supplemental Figure 1C), as determined by an overall increase in CD69 expression on Tregs after infarction in most of the patients (Figure 1D). However, we divided these patients into 2 groups according to their expression levels of CD69 on Tregs: those with high CD69 expression (65% of patients) and those with low CD69 expression (35% of patients) and conducted further analyses (Figure 1D). Patients with high CD69 expression had lower percentages of CD4^+^ T cells and IL-22^+^CD4^+^ T cells but higher percentages of Tregs. In addition, patients with high CD69 expression had more naive and fewer memory Tregs, according to the expression of CD45RA^+^ and CD45RO^+^, respectively (Figure 1E and Supplemental Figure 1D). Unsupervised hierarchical clustering of patients based on myeloid and lymphoid cell types analyzed by FACS (Supplemental Figure 1A) revealed that patients with high CD69 expression had different immune phenotype than did those with low CD69 expression, as they clustered in distinct groups on the *t*-distributed stochastic neighbor embedding (*t*-SNE) plot (Figure 1F).

### CD69 expression improves survival and recovery after LAD ligation in mice.

**Figure 2:**
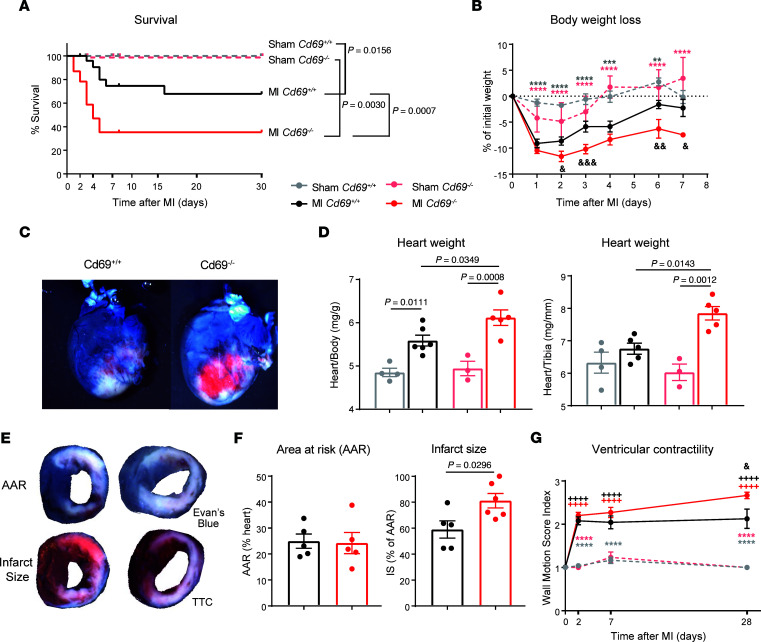
CD69 deficiency worsens heart damage and decreases survival after MI in mice. (**A**) Survival curve of mice after LAD ligation (*n* = 23–29 MI mice, *n* = 11–20 sham-operated mice). Data were pooled from 5 independent experiments and were analyzed by long-rank (Mantel-Cox) test. (**B**) Kinetics of the percentage of body weight loss after LAD ligation (*n* = 9–17 mice). Data represent the mean ± SEM and were analyzed by 2-way ANOVA with Šidák’s multiple-comparison test. (**C**) Representative images of infarcted hearts collected after intravenous injection of Evans blue dye 2 days after surgery. (**D**) Heart weight was normalized to body weight and tibia length 2 days after LAD ligation (*n* = 3–4 sham-operated mice; *n* = 5–6 MI mice). Data are representative of 3 independent experiments and indicate the mean ± SEM. Statistical significance was analyzed by 1-way ANOVA with Tukey’s post hoc test. (**E**) Representative images of heart slices showing the AAR (negative for Evans blue dye) in the upper panels and the extent of necrosis (negative for TTC staining) in the lower panels. (**F**) Histological quantification of the percentage of the LV AAR and the percentage of infarct size (IS) (*n* = 5–6 mice). Data are expressed as the mean ± SEM and were analyzed by unpaired Student’s *t* test. (**G**) Time course of LV dysfunction according to the WMSI measured by echocardiography (*n* = 6–16 MI mice, *n* = 4–8 sham-operated mice). Data were pooled from 3 independent experiments, represent the mean ± SEM, and were analyzed by 2-way ANOVA with Šidák’s multiple-comparison test. Asterisks denote differences between MI and sham-operated mice (light gray for *Cd69^+/+^* mice, light red for *Cd69^–/–^* mice); ampersands denote differences between *Cd69^+/+^* and *Cd69^–/–^* MI mice; plus signs denote differences between each day and day 0. ^&^*P* < 0.05, **^/&&^*P* < 0.01, ***^/&&&^*P* < 0.001, and ****^/++++^*P* < 0.0001.

To evaluate the role of CD69 expression in recovery after MI, we analyzed the survival of mice after ligation of the LAD coronary artery. Survival was significantly reduced in *Cd69^–/–^* mice compared with their wild-type littermates (Figure 2A). *Cd69*-deficient mice had worse recoveries after infarction, as they were unable to reach the baseline weight 1 week after LAD ligation (Figure 2B). In addition, *Cd69^–/–^* mice showed elevated myocardial damage (Figure 2C) and increased heart weight/body weight and heart weight/tibia length ratios 2 days after LAD ligation, indicating that *Cd69^–/–^* hearts may be swollen, perhaps as a consequence of greater inflammation (Figure 2D). The assessment of infarct size and ischemic area at risk by double staining with triphenyltetrazolium chloride (TTC) and Evans blue is a widely used method for the quantification of myocardial damage after infarction (33). In t his study, the infarct size of *Cd69^+/+^* mice ranged from 40%–80 %, in agreement with previous reports in this model (34–36). The absence of CD69 led to a significantly increased infarct size 2 days after infarction, supporting the observation of greater tissue damage in these mice (Figure 2, E and F). Two days after MI, wild-type and CD69-deficient mice had several cardiac rhythm abnormalities, including ventricular premature depolarizations, first-degree atrioventricular block, and ST-segment elevation. However, under cardiac stress conditions induced by isoproterenol injection, the *Cd69^–/–^* mice had an enhancement of the above-mentioned cardiac arrhythmias compared with their wild-type littermates, and, eventually, bradyarrhythmias led to complete heart block, ventricular escape rhythms, and death (Supplemental Figure 2). Evaluation of cardiac dysfunction by echocardiography indicated that the ventricular wall motion score index increased in *Cd69^–/–^* mice as the disease progressed, reaching significance compared with wild-type mice 1 month after infarction, supporting the idea of a worse prognosis in *Cd69^–/–^* mice after LAD ligation (Figure 2G).

Since most patients with MI are eventually reperfused during admission, we analyzed the consequences of CD69 depletion in a mouse model of MI induced by ischemia and reperfusion (I/R). Similar to the data obtained with the permanent LAD ligation model, *Cd69*^–/–^ mice had increased cardiac dysfunction and worse weight recovery after I/R, resulting also in slight decrease in survival (Supplemental Figure 3).

### IL-17^+^ γδT cells are the main source of peripheral IL-17A shortly after MI and are increased in Cd69^–/–^ mice.

**Figure 3:**
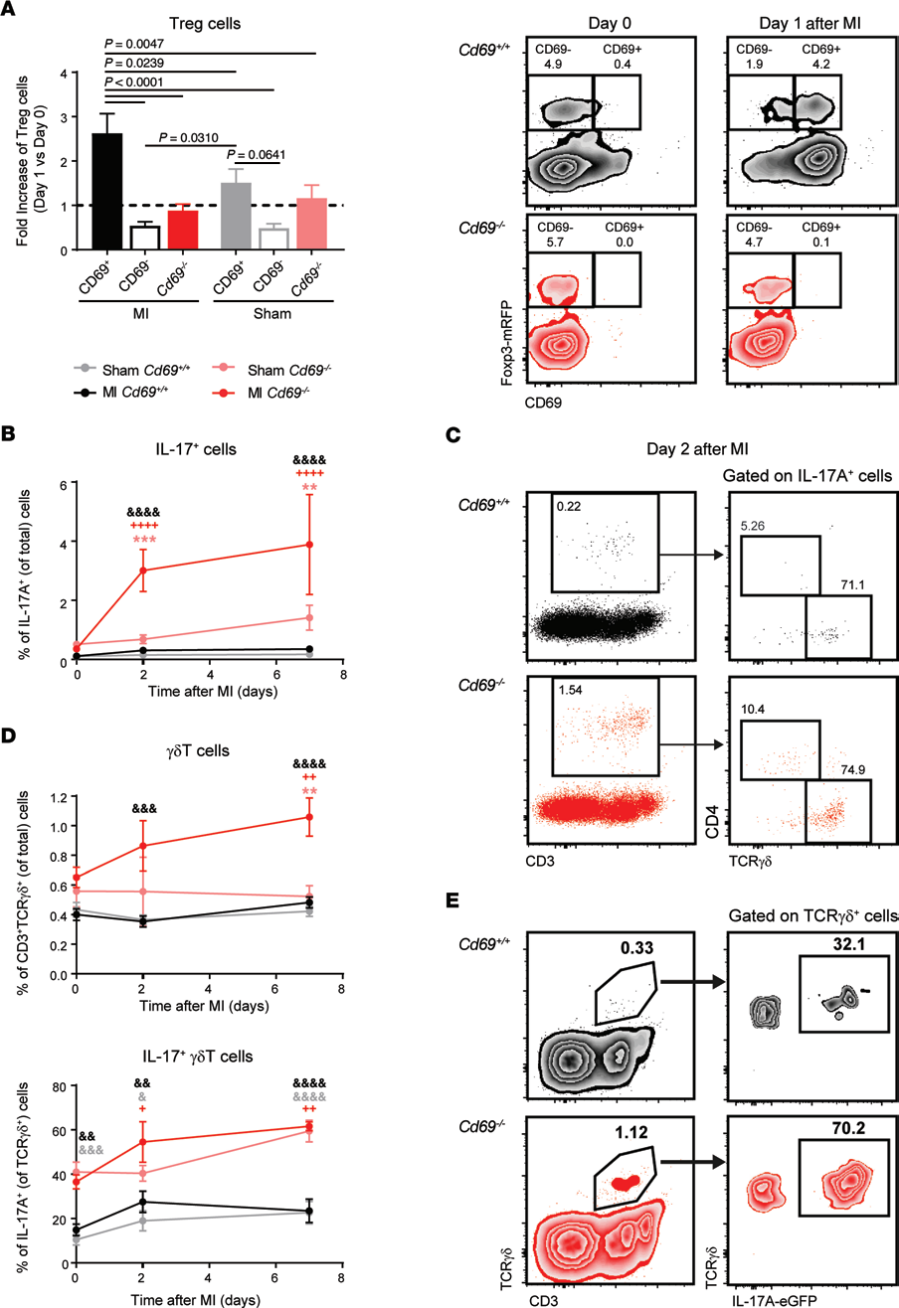
Treg and IL-17A responses in the blood of mice after LAD ligation. (**A**) Fold increase of the percentages of wild-type CD69^+^ Tregs, wild-type CD69^–^ Tregs, and *Cd69^–/–^* Tregs among CD4^+^ cells in peripheral blood 1 day after LAD ligation or sham surgery, compared with the percentages on day 0 (dotted line). Representative density plots of Tregs on day 0 and day 1 after MI are shown on the right (*n* = 10–20). Histograms indicate the mean ± SEM, and data were analyzed by 1-way ANOVA with Tukey’s post hoc test. (**B**) Kinetics of IL-17A^+^ cells in peripheral blood after surgery, expressed as a percentage of total cells. (**C**) Left: Representative dot plots of IL-17A^+^ cells, with the percentages of total cells indicated in the outlined box. Right: Representative dot plots showing the main cell populations positive for IL-17A. (**D**). Kinetics of the percentages of γδT cells and IL-17^+^ γδT cells in peripheral blood after LAD ligation or sham surgery. (**E**) Representative density plots of γδT cells in peripheral blood, with the percentages of cells indicated in the outlined box. Data in **B** and **D** are representative of 4 independent experiments and indicate the mean ± SEM (*n* = 6–10). Statistical significance was analyzed by 2-way ANOVA with Šidák’s multiple-comparison test. Asterisks denote differences between MI and sham-operated mice (light gray for *Cd69^+/+^* mice, light red for *Cd69^–/–^* mice); ampersands denote differences between *Cd69^+/+^* and *Cd69^–/–^* MI mice; plus signs denote differences between each day and day 0. ^+/&^*P* < 0.05, **^/++/&&^*P* < 0.01, ***^/&&&^*P* < 0.001, and ^++++/&&&&^*P* < 0.0001.

It is well known that the number of CD4^+^CD25^+^Foxp3^+^ Tregs increases in the heart during the first day after infarction (37), indicating an antigen-independent migration of Tregs into the injured myocardium. We observed a specific mobilization of CD69^+^ Tregs in wild-type mice after infarction, with a 2.5-fold increase in peripheral blood in the first 24 hours (Figure 3A), mimicking the peripheral response in patients with MI (Figure 1). This mobilization was not observed in either wild-type CD69^–^ or *Cd69^–/–^* Tregs (Figure 3A). Since *Cd69^–/–^* mice have exacerbated Th17 responses in different inflammatory diseases (38), we analyzed the kinetics of IL-17A^+^ cells in the blood 1 week after infarction. We found that *Cd69^–/–^* mice had a significantly increased IL-17 response during the first 2–7 days after LAD ligation, which was not observed in *Cd69^+/+^* mice (Figure 3B). Most IL-17A–producing cells in the peripheral blood after infarction are CD3^+^ T cells (Figure 3C), with the majority of them being γδT cells, which are the main source of IL-17 in the blood after infarction, instead of Th17 cells (Figure 3C). *Cd69^–/–^* γδT cells expressed higher levels of IL-17A even in steady state, and *Cd69*^–/–^ mice had an amplified IL-17^+^ γδT cell response in peripheral blood shortly after MI (Figure 3, D and E). As IL-17^+^ γδT cells are well known as initiators of inflammation (39) and induce apoptosis of cardiac myocytes (40), we postulate that this population may contribute to the increased damage observed in *Cd69^–/–^* mice after LAD ligation.

### IL-17^+^ γδT cells rapidly accumulate in the infarcted myocardium in Cd69^–/–^ mice.

**Figure 4:**
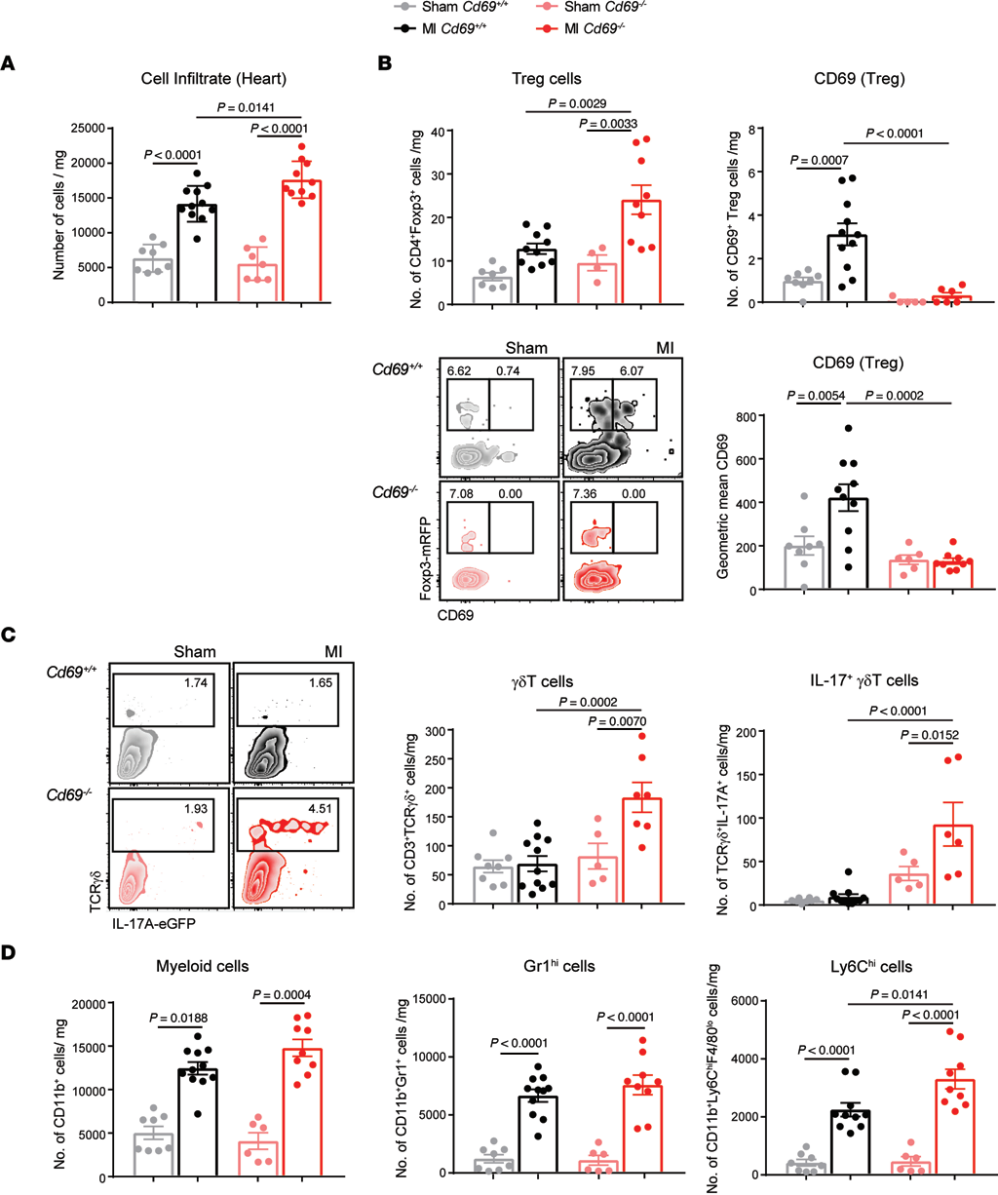
Myocardial accumulation of CD69^+^ Tregs and Il-17^+^ γδT cells after LAD ligation. (**A**) Leukocyte cell numbers per milligram of heart tissue in the myocardium 2 days after infarction. (**B**) Quantification of the number of Tregs (CD4^+^Foxp3^+^) and CD4^+^Foxp3^+^CD69^+^ cells per milligram of heart tissue and CD69 mean fluorescence expression on Tregs in the heart. Representative density plots showing gating on CD45^+^CD11b^–^CD4^+^ cells. (**C**) Representative density plots gated on CD45^+^CD11b^–^CD3^+^ cells and numbers of γδT cells and Il-17^+^ γδT cells per milligram of tissue. (**D**) Quantification of total cell numbers per milligram of CD11b^+^ myeloid cells, CD11b^+^Gr1^hi^ cells, and CD11b^+^F4/80^lo^Ly6C^hi^ cells in the heart. Heart cell–infiltrating populations were evaluated 2 days after infarction (*n* = 6–11 animals per group). Data are representative of 4 independent experiments and indicate the mean ± SEM. Statistical significance was analyzed by 1-way ANOVA with Tukey’s post hoc test. *P* values for significant differences are shown.

Next, we analyzed the leukocyte populations infiltrating the myocardium to characterize inflammation in infarcted tissue from *Cd69^–/–^* mice. Quantification of the total number of leukocytes per milligram of infarcted tissue showed that the hearts of *Cd69^–/–^* mice exhibited increased inflammation on day 2 after MI compared with their *Cd69^+/+^* littermates (Figure 4A). Consistent with previous studies, we found that Tregs were recruited to the heart after ischemia. However, we detected an increased number of CD69^+^ Tregs and higher CD69 expression on Tregs in the myocardium of MI mice versus that of sham-operated mice, indicating a selective migration of these cells to the myocardium after LAD ligation (Figure 4B). The analysis of CD69 expression in different cardiac cell populations revealed that CD45^–^CD31^+^ endothelial cells, CD45^+^CD11b^+^ myeloid cells, and CD45^+^CD11b^–^CD4^+^Foxp3^–^ T effector cells did not have upregulated expression of CD69 after MI to the same extent as Tregs (Supplemental Figure 4, A and B). These data support a dominant role of CD69 in the Treg compartment after MI. In the infarcted myocardium, γδT cells peak 1 week after ischemia and are the main producers of IL-17 (23). Indeed, we confirmed that the majority of IL-17A–producing cells were TCRγδ^+^ cells (~75%) in both *Cd69^+/+^* and *Cd69^–/–^* mice, although γδT cell and IL-17^+^ γδT cell infiltration into the myocardium was significantly increased in *Cd69^–/–^* mice as early as 2 days after MI (Figure 4C). Neither *Cd69^+/+^* nor *Cd69^–/–^* mice showed a significant recruitment of Th17 cells at this early time point (Supplemental Figure 4C). Furthermore, although we observed no differences in the number of CD11b^+^ myeloid cells or CD11b^+^Gr1^hi^ granulocytes between genotypes, *Cd69^–/–^* mice had a higher number of inflammatory Ly6C^hi^ cells accumulated in the infarcted myocardium (Figure 4D and Supplemental Figure 4D), indicating a more proinflammatory phenotype (41). In parallel, the pathogenic IL-17^+^ γδT cell population was significantly increased in the mediastinal lymph nodes draining the heart after infarction in *Cd69^–/–^* mice (Supplemental Figure 4E).

### CD69^+^ Tregs induce apoptosis and reduce IL-17A production in γδT cells in a CD39-dependent manner in mice.

**Figure 5:**
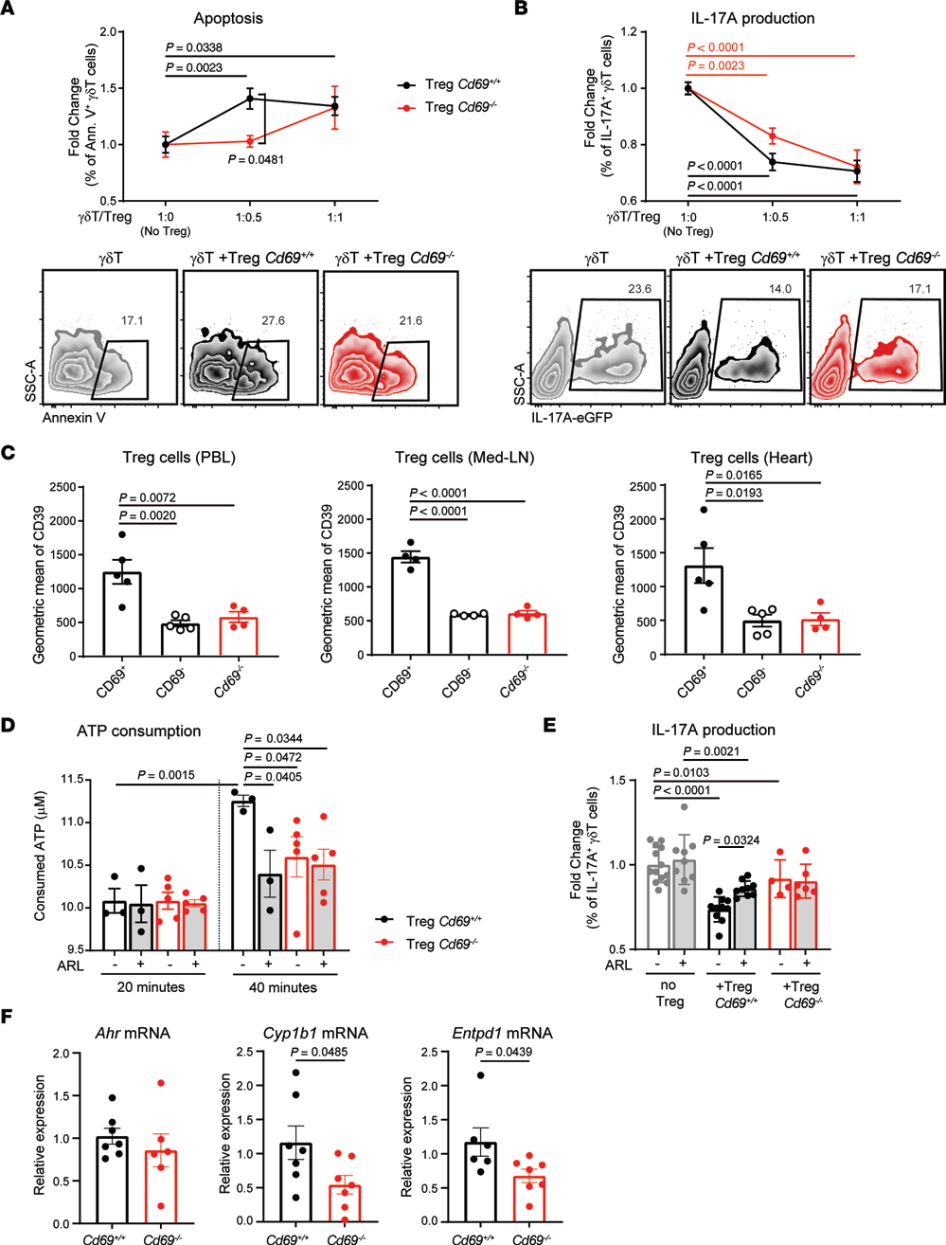
Expression of CD39 by CD69^+^ Tregs after MI mediates the inhibition of γδΤ cells. (**A**) Sorted wild-type γδT cells were cocultured for 24 hours with *Cd69^+/+^* or *Cd69^–/–^* sorted Tregs at the indicated γδT/Treg ratios. Apoptosis of γδT cells, represented as the fold increase of annexin V^+^ (Ann. V^+^) γδT cells versus γδT cells alone (ratio 1:0) (*n* = 4–10). A representative zebra plot of the 1:0.5 ratio, gated on γδT cells is shown. (**B**) Inhibition of IL-17A production by γδT cells, plotted as the fold change of IL-17A^+^ γδT cells for each ratio versus the 1:0 ratio (*n* = 6–12). Representative zebra plots of the 1:0.5 ratio, gated on γδT cells, are shown. Data in **A** and **B** were pooled from 4 independent experiments. Data indicate the mean ± SEM. Statistical significance was determined by 2-way ANOVA with Sidak’s multiple-comparison test. Significant *P* values are shown (black for *Cd69^+/+^* Tregs and red for *Cd69^–/–^* Tregs). (**C**) CD39 expression on Tregs in PBLs, mediastinal lymph nodes (Med-LN), and heart was measured by FACS, 2 days after MI (*n* = 4–5). Data indicate the mean ± SEM. Statistical significance was determined by 1-way ANOVA with Tukey’s post hoc test. (**D**) Extracellular ATP was measured in the supernatant of isolated Tregs, in the presence or absence of ARL 67156 (ARL) at the indicated time points after ATP supplementation (*n* = 3–5). Data are from 1 representative independent experiment of 4 experiments and indicate the mean ± SEM. Statistical significance was determined by mixed-effects 2-way ANOVA with Šidák’s multiple-comparison test. (**E**) IL-17A production by sorted γδT cells in the presence of Tregs and/or ARL 67156, fold change versus the percentage of IL-17A^+^ γδT cells alone (*n* = 3–7). Data are representative of 3 independent experiments and indicate the mean ± SEM. Statistical significance was determined by 2-way ANOVA with Šidák’s multiple-comparison test. (**F**) Quantification of *Ahr*, *Cyp1b1*, and *Entpd1* mRNA levels in Tregs by qPCR (*n* = 6–7). Data were pooled from 2 independent experiments and represent the mean ± SEM. Statistical significance was determined by unpaired Student’s *t* test.

Previous studies provide evidence for antigen-independent inhibition of γδT cells by Tregs (42, 43), although the mechanisms remain poorly understood. Our data suggest that CD69 expression on Tregs may be involved in limiting γδT cell activity. To test this hypothesis, we cocultured sorted *Cd69^+/+^* or *Cd69^–/–^* Tregs with *Cd69^+/+^* γδT cells from peripheral lymph nodes (Supplemental Figure 5, A and B). Our data indicate that CD69^+^ Tregs induced apoptosis in γδT cells more efficiently than did *Cd69^–/–^* Tregs (Figure 5A). Both *Cd69^+/+^* and *Cd69^–/–^* Tregs successfully decreased IL-17A production in a dose-dependent manner. However, at lower numbers of Tregs, *Cd69^+/+^* Tregs showed a more significant reduction of IL-17A production (Figure 5B).

CD39 is a membrane ectonucleotidase expressed in most Tregs that, together with CD73, transforms extracellular ATP into adenosine (44, 45). It has been shown that CD39 on Tregs can mediate the inhibition of innate (46) and nonimmune cells (47) by immunosuppression-independent mechanisms upon tissue injury. We evaluated whether the observed inhibition of γδT cells by CD69^+^ Tregs after infarction was mediated by CD39. Our data show that CD69^+^ Tregs expressed higher levels of membrane CD39 than did CD69^–^ or *Cd69^–/–^* Tregs in peripheral blood, mediastinal lymph nodes, and cardiac infiltrate 2 days after MI (Figure 5C). Sorted *Cd69^+/+^* Treg also showed an increase in extracellular ATP consumption compared with *Cd69^–/–^* Tregs. The addition of ARL 67156, a chemical inhibitor of CD39, reduced ATP consumption of *Cd69^+/+^* Tregs and abolished the differences between genotypes (Figure 5D). These results support the idea that CD39 activity was impaired in CD69-deficient Tregs. Regarding the observed reduction of IL-17A production in γδT cells by CD69^+^ Tregs (Figure 5B), it was reverted in the presence of the inhibitor ARL 67156, whereas no effect was observed on *Cd69^–/–^* Tregs (Figure 5E). A direct effect of ARL 67156 on γδT cells was ruled out, since no γδT cell inhibition was observed when ARL 67156 was added in the absence of Tregs.

The aryl hydrocarbon receptor (AhR) is one of the main factors that mediates CD39 expression. Ligand-dependent activation of the AhR induces the expression of *Entpd1* (CD39-encoding gene) through direct binding to XRE regions of the *Entpd1* promoter (48). Our data also validated that *Entpd1* mRNA levels, as well as CD39 activity, were reduced in *Ahr^–/–^* Tregs, supporting the notion that AhR is a dominant transcription factor for *Entpd1* expression in this cell type (Supplemental Figure 5, C and D). We have previously described that CD69 allows the uptake of AhR ligands such as l-tryptophan in T cells by association with the aromatic–amino acid transporter complex on the membrane, allowing AhR activation (49). We observed that Tregs lacking CD69 exhibited decreased expression of AhR transcriptional targets, including *Cyp1b1* and, importantly, *Entpd1* (Figure 5F). Additionally, AhR-deficient CD69^+^ Tregs exhibited decreased CD39 expression compared with wild-type Tregs (Supplemental Figure 5E), supporting the idea that AhR signaling mediates CD39 expression in CD69^+^ Tregs. Together, these data suggest that CD69 expression on Tregs allowed AhR-mediated expression of CD39, increasing ATP conversion to adenosine, which triggered the inhibition of pathogenic IL-17^+^ γδT cells and controlled inflammation after MI.

### Adoptive transfer of CD69-sufficient Tregs into Cd69^–/–^ mice improves survival and reduces inflammation and cardiac damage after MI.

**Figure 6:**
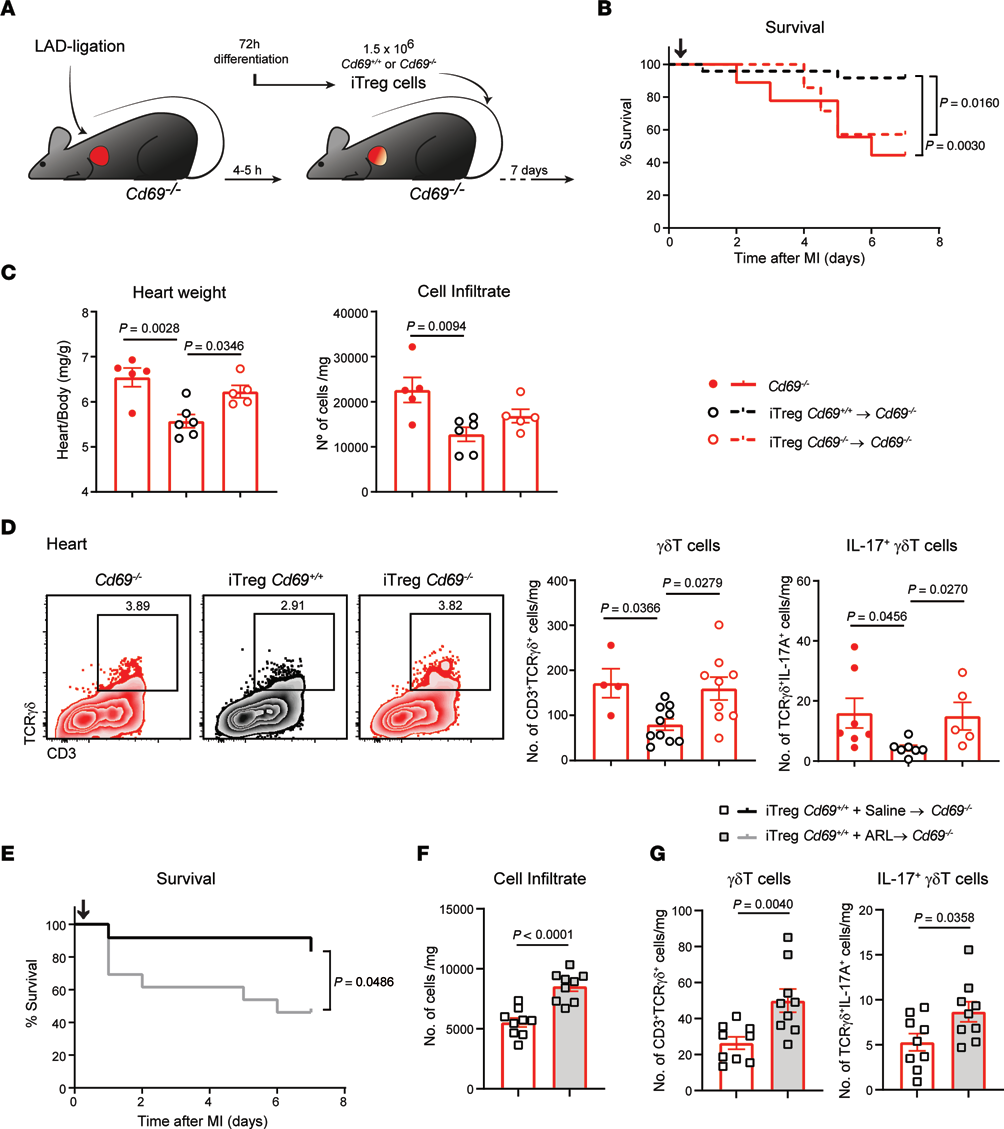
Adoptive transfer of CD69-sufficient Tregs into *Cd69^–/–^* mice reduces myocardial inflammation and improves survival after MI. (**A**) Schematic workflow of the iTreg adoptive transfer after LAD ligation. (**B**) Survival after LAD ligation (*n* = 7–24). Black arrow depicts the time of iTreg inoculation (4–5 hours after infarction). *Cd69^–/–^* mice without cell transfer were used as controls. The *P* value was calculated by long-rank (Mantel-Cox) test. (**C**) Heart/body weight ratio and total leukocyte numbers per milligram of heart tissue 7 days after LAD ligation (*n* = 5–6). (**D**) Representative density plots (gated on CD45^+^CD11b^–^ cells) and numbers of γδT cells and IL-17^+^ γδT cells per milligram of myocardial tissue. Data in **C** and **D** correspond to 1 representative independent experiment of 3 experiments. Data indicate the mean ± SEM and were analyzed by 1-way ANOVA with Tukey’s post hoc test. (**E**) Survival after LAD ligation (*n* = 12–13), *Cd69^+/+^* iTreg transfer, and ARL 67156 or saline administration. Black arrow depicts the time of iTreg inoculation (4–5 hours after infarction). The *P* value was calculated by long-rank (Mantel-Cox) test. (**F**) Total leukocyte numbers per milligram of heart tissue 7 days after LAD ligation (*n* = 9). (**G**) Numbers of γδT cells and IL-17^+^ γδT cells per milligram of myocardial tissue (*n* = 9). Data in **F** and **G** were pooled from 4 independent experiments and indicate the mean ± SEM. Statistical significance was determined by unpaired Student’s *t* test.

Next, we performed adoptive transfer experiments to assess the specific role of CD69 on Tregs in the control of inflammation and recovery after infarction. In vitro–differentiated Tregs (iTregs) from naive *Cd69^+/+^* and *Cd69^–/–^* CD4^+^ T cells (Supplemental Figure 6A) were injected intravenously into *Cd69^–/–^* mice 4–5 hours after LAD ligation, and mice were monitored for 1 week (Figure 6A). Therapy with *Cd69^+/+^* iTregs improved survival of *Cd69^–/–^* mice after infarction (Figure 6B). Furthermore, the heart/body weight ratio was preserved in the *Cd69^–/–^* mice transferred with *Cd69^+/+^* iTregs, as was the total number of leukocytes infiltrating the myocardium (Figure 6C). CD69 expression drives CD39 expression in myocardial Treg and T effector cells, although CD69^+^ Tregs exhibited the highest CD39 levels (Supplemental Figure 6B). Interestingly, infiltrating IL-17A^+^ γδT cells were reduced in the myocardium after transfer of *Cd69^+/+^* iTregs but not *Cd69^–/–^* iTregs, supporting CD69-mediated inhibition of this population by Tregs (Figure 6D). In parallel, myeloid cells, including proinflammatory Ly6C^hi^ monocytes, were also reduced (Supplemental Figure 6D). All these data suggest that CD69-expressing Treg transfer reverted the immune-mediated cardiac damage after MI in *Cd69^–/–^* mice. Interestingly, when ARL 67156 was administered in vivo to inhibit CD39, *Cd69^+/+^* iTregs lost their ability to restore survival, myocardial inflammation, and IL-17A^+^ γδT cell accumulation in the heart (Figure 6, E–G), suggesting that the therapeutic effects of *Cd69^+/+^* iTreg transfer was CD39 dependent. Transfer of *Cd69^+/+^* iTregs into wild-type infarcted mice (Supplemental Figure 7A) also improved myocardial inflammation and survival to a greater extent than did the transfer of *Cd69^–/–^* iTregs, although without reaching significance, probably due to the presence of endogenous CD69^+^ Tregs (Supplemental Figure 7, B and C). Probably for the same reason, we found no differences in myocardial γδT cells in the *Cd69^+/+^* iTreg–transferred wild-type mice (Supplemental Figure 7D).

### Early expression of CD69 on Tregs is associated with a lower risk of HF development in patients with MI.

**Figure 7:**
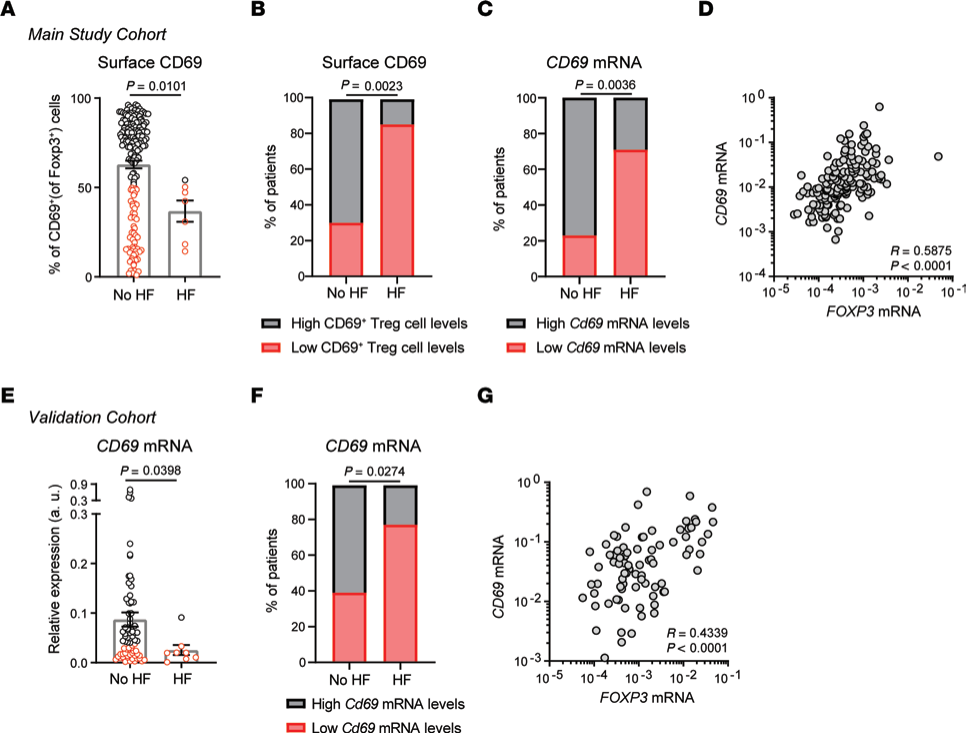
High CD69 expression in patients early after MI is associated with a decreased risk of developing HF. (**A**) After 2.5 years of clinical follow-up, patients from the main study cohort were stratified according to whether or not they developed HF. CD69 expression on Tregs at the time of hospital admission for acute MI in patients who developed HF (*n* = 7) or did not develop HF (*n* = 180). Data were analyzed by Mann-Whitney *U* test. (**B**) Percentage of patients with low or high levels of surface CD69 expression on Tregs, measured by FACS in the main study cohort. The *P* value was calculated using a χ^2^ test. (**C**) Frequency of patients with low or high levels of *CD69* mRNA expression, measured by qPCR in the main study cohort (the mean normalized *CD69* 2^–ΔCt^ values were used to discriminate patients expressing low or high levels of *CD69*). (**D**) Correlation of *FOXP3* and *CD69* mRNA expression in PBLs from individuals in the main study cohort. Spearman’s correlation coefficient (*r*) and *P* values are shown. (**E**) *CD69* mRNA levels measured by qPCR in total PBLs from the independent validation cohort of patients (*n* = 75 with no HF and *n* = 9 with HF). Data were analyzed by Mann-Whitney *U* test. (**F**) Frequency of patients with low or high levels of *CD69* mRNA, measured by qPCR in the validation cohort (the mean normalized *CD69* 2^–ΔCt^ values were used to discriminate patients with low or high *CD69* expression). The *P* value was calculated using a χ^2^ test. (**G**) Correlation of *FOXP3* and *CD69* mRNA expression in PBLs from individuals in the validation cohort. Spearman’s correlation coefficient (*r*) and *P* values are shown.

We tested the prognostic value of CD69 expression after MI in those patients who completed follow-up at the time this manuscript was written. The mean clinical follow-up time of our primary study population was 2.5 years (Supplemental Table 3). CD69 expression on Tregs, measured at the time of hospital admission during the index MI, was lower in patients who were subsequently rehospitalized for HF (Figure 7A). Interestingly, the LV ejection fraction, but not CK or troponin T levels, also decreased during MI in the patients who subsequently developed HF (Supplemental Figure 8A). After stratifying patients according to high or low CD69 expression, as in Figure 1D, we observed that most patients who developed HF within the first 2.5 years of MI belonged to the group expressing low membrane CD69 levels on Tregs (Figure 7B). Determination of *CD69* mRNA expression by quantitative PCR (qPCR) in total peripheral blood leukocytes (PBLs) also revealed an association with disease outcome, as most of those patients expressing low *CD69* mRNA levels developed HF (Figure 7C). *CD69* mRNA levels correlated with surface CD69 levels, associating CD69 protein and transcript expression (Supplemental Figure 8B), and with *FOXP3* mRNA levels, highlighting a predominant CD69 expression by Tregs (Figure 7D). The equivalence in measuring CD69 by qPCR or by FACS supports the validity of using either method for the prediction of HF development. Importantly, the percentage of CD69^+^ Tregs remained as an independent predictor of the development of HF after adjusting for the levels of cardiac damage, age, and sex (Table 1).

In parallel, we prospectively analyzed the association of early CD69 expression (during the first 24 hours of hospitalization for MI) with clinical outcome after MI in an additional independent validation cohort of 84 patients with similar follow-up times (Supplemental Tables 4 and 5). Quantification of *CD69* mRNA levels in frozen PBLs confirmed that patients who developed HF expressed lower *CD69* levels at acute MI (Figure 7, E and F). Although data on CD69 expression on the surface of Tregs were not available in this cohort, *CD69* mRNA expression correlated significantly with *Foxp3* expression, establishing an association between CD69 expression and the Treg subset, as in the main patient cohort (Figure 7G). Thus, these data support the idea that CD69 expression during acute MI is associated with a lower risk of developing HF after MI.

## Discussion

This study identifies CD69 as a key Treg receptor for controlling immune-mediated myocardial damage early after MI. We observed a specific deployment of CD69^+^ Tregs in the peripheral blood of MI patients and LAD ligation mice. Moreover, CD69-deficient mice and patients with low CD69 levels shortly after acute MI had a worse prognosis after the ischemic event.

The general idea is that Tregs are recruited to the myocardium during the first few days after MI to control excessive inflammation and prevent cardiac deterioration (7, 9, 10, 37, 50). We have recently shown that CD69 expression on lymphocytes prevents atherosclerosis progression in mice and that low CD69 levels in PBLs predict subclinical atherosclerosis in asymptomatic individuals after adjustment for traditional cardiovascular risk factors (18). Here, we describe for the first time to our knowledge that CD69 expression is pivotal for the antiinflammatory properties of Tregs after ischemic myocardial damage. CD69 expression in T cells is an inflammatory brake that promotes Treg differentiation and suppressor function and prevents proinflammatory T cell responses in multiple disease scenarios (38). In this study, we describe a mechanism by which CD69 expression on Tregs promoted antigen-independent inhibition of proinflammatory γδT cells after MI. CD69 induced AhR-dependent CD39 expression and extracellular ATP conversion to adenosine, inhibiting effector cytokine production and inducing apoptosis in γδT cells infiltrating the ischemic myocardium.

Mice lacking CD69 showed increased myocardial inflammation and dysfunction, leading to a rapid decrease in survival during the first week after infarction. Therapy with CD69^+^ Tregs in the first hours after LAD ligation reduced myocardial inflammation and improved the survival of *Cd69^–/–^* mice, indicating that CD69 expression specifically on the Treg subset was sufficient to alleviate the recovery after MI.

Acute MI is the leading cause of mortality worldwide, with HF secondary to MI as one of the most important complications (51). In a follow-up study of 2 independent clinical cohorts, we found that patients who were rehospitalized because of HF had lower CD69 expression levels within the first hours of acute MI. Even after adjusting for age, sex, and the level of cardiac damage, CD69 expression on Tregs remained as an independent predictor of HF development after MI. Thus, in agreement with the phenotype observed in mice, CD69 expression during MI was associated with a better clinical outcome in these patients. Although these results have been validated in 2 independent cohorts with multivariate adjustment, the number of patients who developed HF was small. Therefore, our findings should be reproduced in larger cohorts, ideally including a longer post-MI time interval.

In order to shed light on the regulatory mechanism of CD69 after MI, we explored the inflammatory signature in a mouse model of LAD ligation. We found that the absence of CD69 resulted in a rapid and detrimental excess of inflammatory infiltrating leukocytes in mice as early as 2 days after LAD ligation. Increased numbers of IL-17^+^ γδT cells characterize the cellular heart infiltrate of *Cd69^–/–^* mice, being the main source of IL-17A early after MI. The increased recruitment of IL-17^+^ γδT cells could explain the worse prognosis of *Cd69^–/–^* mice after MI, as γδT cells induce cardiomyocyte apoptosis (40), and their production of IL-17 promotes fibrosis, sustains neutrophil/monocyte infiltration, and polarizes macrophages toward a proinflammatory phenotype (23). We observed that peripheral *Cd69^–/–^* γδT cells expressed markedly higher levels of IL-17A under basal conditions, suggesting that CD69 may also be playing a role in the generation of this T cell subset. We found that CD69^+^ Tregs were substantially recruited to the infarcted myocardium, but *Cd69^–/–^* mice showed an even higher total number of Tregs in the myocardium at this time point, probably due to a parallel compensatory antiinflammatory response to balance the tissue damage.

Interestingly, the accumulation of IL-17^+^ γδT cells in the myocardium was impaired when *Cd69^+/+^*, but not *Cd69^–/–^*, Tregs were transferred, suggesting that Tregs inhibited IL-17^+^ γδT cells by a mechanism involving CD69. It is well established that Tregs suppress effector CD4^+^ T cells and CD8^+^ T cells (52, 53). However, these cell populations play a minor a role after MI. It has been described that Tregs can reduce the proliferation of human phosphoantigen–expanded γδT cells (43) and the proliferation and cytokine production of murine intestinal γδT cells (42) in an antigen-independent manner, suggesting that Tregs are also capable of inhibiting this T cell subset. Our data show that mouse γδT cells underwent apoptosis and decreased IL-17A production in vitro in the presence of Tregs, indicating an inhibitory effect of Tregs on γδT cells. This effect was dose dependent and was mediated by CD69, as *Cd69^–/–^* Tregs showed a lower inhibitory capacity. These results may explain the decrease in the number of IL-17A^+^ γδT cells in the infarcted myocardium after the adoptive transfer of *Cd69^+/+^* Tregs.

In order to further explore the molecular basis of this rapid inhibition of γδT cells, we examined the CD39-mediated, antigen-independent mechanisms of Treg suppression. CD39 is an ectonucleotidase that hydrolyzes extracellular ATP, released from damaged tissue, to AMP, which is further degraded to adenosine by CD73. Uptake of adenosine by innate and T effector cells promotes apoptosis and cell inhibition (45, 46), but uptake by Tregs induces antiinflammatory properties (54). Tregs from patients with MI overexpress CD39 early after revascularization (10). Our work shows that CD69^+^ Tregs infiltrating the peripheral blood, draining lymph nodes, and heart expressed higher levels of membrane CD39 than did CD69^–^ or *Cd69^–/–^* Tregs 2 days after MI. Since CD69 facilitates the entrance of AhR ligands (49), the increased expression and activity of CD39 (a transcriptional target of AhR) in CD69-sufficient Tregs could be explained by the observed increased activation of the AhR pathway. In agreement, different studies described that CD39 confers protection after infarction, showing that CD39 overexpression ameliorates the progression of myocardial damage after MI (55–57) or that *Cd39^–/–^* mice develop exacerbated cardiac damage (58). Moreover, adoptive transfer of *Cd39^–/–^* iTregs fails to prevent inflammation and achieve cardiac protection after MI (10). Thus, Tregs deficient in either CD69 or CD39 present similar dysfunctional properties early after MI. Consistently, we report that chemical inhibition of CD39 in vitro and in vivo resulted in impaired suppression of γδT cells by CD69-sufficient Tregs, suggesting that CD69^+^ Tregs exert their inhibition of γδT in a CD39-dependent manner. All this evidence suggests a novel molecular mechanism of Treg protection against inflammation-mediated cardiac impairment, although further exploration in a clinical setting is needed.

In conclusion, we have identified CD69 as a crucial orchestrator of the cardioprotective function of Tregs after MI. Although additional studies are needed to prospectively confirm the diagnostic and therapeutic value of circulating CD69^+^ Tregs, we believe this target opens new, specific cellular avenues for precision medicine to improve the management of patients with MI.

## Methods

### Study design.

The overall objective of our study was to investigate the relevance of CD69-expressing Tregs in the progression of myocardial damage after MI. For this purpose, we analyzed the expression of CD69 on Tregs in peripheral blood from patients with MI. In addition, to further elucidate the mechanism of action of these cells, we performed permanent LAD ligation to induce MI in *Cd69^–/–^* mice and their *Cd69^+/+^* littermates. Peripheral immune response and heart inflammation, necrosis, and function were evaluated. *Cd69^–/–^* infarcted mice were also transferred with CD69-sufficient iTregs to narrow down the protective effect of CD69 to the Treg compartment. Further in vitro experiments adding Tregs to γδT cell cultures were performed to prove a direct CD69-dependent inhibitory effect on γδT cells.

The genotyping of mice was recorded throughout the entire period of the study. Mice were randomly assigned to each experimental group, and the only criteria used to stratify them was the genotype. Infarct size quantification was performed in a blinded manner. For survival plots, mice that died during the surgical procedure were excluded. Animals were excluded that had undetectable/marginal infarct zones by echocardiography 2 days after surgery, a result that was probably due to an unsuccessful LAD ligation. For all experiments, sample sizes were determined by our previous data from similar experiments and prior literature to ensure statistical relevance. At least 3 biological replicates were used for each assay, and each experiment was performed at least 3 times when applicable.

All blood samples from patients suspected of meeting all the MI inclusion criteria were processed in a blinded manner upon hospital admission of the patient, during the study period. Only patients who had a confirmed MI after extensive diagnosis and met the inclusion criteria were included in the analyses. Data from patients with an uncertain diagnosis were excluded. Blood samples from patients processed more than 24 hours after extraction were also excluded from the study, since degradation of blood components might have altered the sample. The number of human samples was determined according to an estimation of clinical relevance. The incidence of HF and death was assessed in the patients with MI for whom FACS was measured and a follow-up was available at the time of the writing of this manuscript. A similar number of healthy volunteers with normal ECG and heart function measured by echocardiography were included in the study as comparators.

### Human blood sample collection included in the study.

Blood samples from patients with MI were prospectively collected from the arterial sheath used for coronary angiography and percutaneous coronary intervention prior to the administration of heparin, between March 2017 and May 2019. All demographic, clinical, angiographic, and procedural characteristics of these patients with MI were also prospectively collected. Revascularization and subsequent clinical management in the coronary care unit and in the cardiology ward were performed following the guideline recommendations for MI (30) (Supplemental Tables 1 and 3). All patients with MI were systematically followed at a dedicated outpatient clinic.

In parallel, we analyzed blood samples from 80 healthy donors without any cardiac disease, as assessed by normality of cardiac markers, electrocardiography, and echocardiography.

The validation cohort consisted of a consecutive series of patients admitted for MI to the Hospital de la Santa Creu i Sant Pau, Barcelona, Spain. Baseline clinical characteristics, clinical presentation, and in-hospital evolution are summarized in Supplemental Table 4. All patients were followed, and the predefined adverse clinical outcomes (HF and all-cause death) are summarized in Supplemental Table 5. Blood samples were collected from patients within the first 24 hours of their hospital admission and prior to heparin administration.

### Mice.

CD69-deficient mice were generated as described previously (15). Wild-type or CD69-deficient mice used for experiments were 8- to 12-week-old males on a C57Bl/6 background. Foxp3-RFP/IL17-eGFP reporter mice were provided by R.A. Flavell (Yale University, New Haven, Connecticut, USA) and crossed with *Cd69^+/+^* or *Cd69^+/+^* mice. Different experimental groups were sex and age matched with the offspring of littermate mice. Animals were housed and used under specific pathogen–free (SPF) conditions at the CNIC animal facility.

### Mouse model of MI.

For MI induction, a permanent ligation of the LAD coronary artery was performed. Briefly, mice were anesthetized with sevoflurane (4%) and intubated using a 24 gauge catheter with a blunt end. Mice were artificially ventilated with a mixture of O_2_ and air (1:1, vol/vol) using a rodent ventilator (Minivent 845, Harvard) with 160 strokes/minute in a total volume of 250 μL. Mice were placed on a heating pad to maintain body temperature at 37°C. A thoracotomy was performed through the fourth left intercostal space, the pericardium was opened, and the heart was exposed. The proximal LAD coronary artery was localized and permanently ligated by passing a 7-0 silk suture around the artery. Sham-operated mice were analyzed in parallel as surgical controls.

### Mouse infarct size quantification.

Animals were anesthetized and 0.5 mL of 1% (w/v) Evans blue dye was infused intravenously through the vena cava 36 hours after surgery. We rapidly opened the thoracic cavity to perfuse hearts with PBS and washout the excess of Evans blue dye. The heart was then harvested and rinsed, and the atria were removed. The hearts were photographed before being cut into transverse slices (*n* = 4–5 per ventricle). Then, slices were weighed. The palish negative area for Evans blue delineated the area at risk (AAR), i.e., myocardium lacking blood flow. In order to differentiate infarcted from viable tissue, the same slices were incubated in TTC (1% [w/v] in PBS) at 37°C for 15 minutes. The sections were then rephotographed and weighed. After the incubation with TTC, Evans blue staining cleared out, and the sections showed 2 areas: 1 necrotic (palish negative for TTC staining: infarcted myocardium) and 1 alive (red positive for TTC staining). Regions negative for Evans blue staining (AAR) and for TTC (infarcted myocardium) were quantified in a blinded manner using ImageJ with FIJI image processing package (NIH). The percentages of AAR and infarcted myocardium were weighted and corrected independently for each slice, and the total milligrams of AAR and TTC-negative regions was calculated for each heart. The AARs were determined according to the mg/mg ratio of AAR/ventricle weight, and the infarct size was determined by the infarcted myocardium/AAR ratio.

### Electrocardiography in mice.

Two days after LAD ligation, mice were anesthetized with 0.5%–2% isoflurane in oxygen, administered via the nose cone, with adjustment of isoflurane delivery to maintain the heart rate around 500 ± 50 bpm. Surface ECGs were obtained by using unipolar and bipolar limb leads for 5 minutes under basal conditions (Biopac Systems). Then, 1.5 mg/kg isoproterenol (MilliporeSigma) was injected intraperitoneally, and ECGs were monitored for another 15–25 minutes. ECGs were blindly analyzed by an expert using Acknowledge, version 4.1.1, for MP36R (BIOPAC Systems).

### Echocardiography in mice.

Transthoracic echocardiography was performed in a blinded manner by an expert operator using a high-frequency ultrasound system (Vevo 2100, Visualsonics) with a 30 MHz linear probe. 2D and M-mode echocardiography were performed at a frame rate of more than 230 frames/second. Mice were lightly anesthetized with 0.5%–2% isoflurane in oxygen, adjusting the isoflurane delivery to maintain the heart rate at 450 ± 50 bpm. Mice were placed in the supine position and maintained at normothermia using a heating platform and warmed ultrasound gel. A base apex ECG was continuously monitored. Longitudinal and short-axis views of the left ventricle were acquired at the papillary muscle level for M-mode and also medium and apical levels for 2D. For the analysis of the wall motion score using echocardiography, the regional LV function was evaluated in the parasternal long-axis view. The LV wall was subdivided into 6 segments (basal, mid, and apical in the anterior and posterior walls). Each segment was scored in a blinded manner by an independent evaluator on the basis of its motion and systolic thickening, using the following guidelines of the American Society of Echocardiography (59): (a) normal or hyperkinetic; (b) hypokinetic (reduced thickening); (c) akinetic (absent or negligible thickening, e.g., scar); and (d) dyskinetic (systolic thinning or stretching, e.g., aneurysm). The number of dysfunctional segments was quantified, and the total wall motion score index (WMSI) representing the sum of the score of the 6 individual segments in each heart was calculated.

### Isolation of infiltrating cells from mouse heart.

Hearts were perfused with 10 mL PBS and removed from the chest cavity. The hearts were then minced and digested with collagenase IV (100 U/mL; Gibco, Thermo Fisher Scientific) for 45 minutes at 37°C under constant agitation. The resulting cell suspensions were filtered through 40 μm cell strainers (BD Falcon) and washed twice with PBS, 0.5% BSA, and 1 μM EDTA. Erythrocytes were removed using hypotonic buffer, and the number of leukocytes was assessed. Single-cell suspensions were stained as described below, and the different cell populations were analyzed by FACs.

### FACS analysis.

PBLs were isolated from mouse or human blood samples using Ficoll-Isopaque (density = 1.121 g/mL) gradient centrifugation. Human/mouse PBLs or single-cell suspensions of mouse lymph nodes or heart-infiltrating leukocytes were incubated in PBS plus 0.05% BSA plus 0.01% EDTA buffer with fluorochrome-conjugated antibodies. PBLs were stained in fresh for myeloid cell markers and cultured overnight for bystander activation in plates coated with 3 μg/mL purified anti-CD3 antibody (for human cells: ΟΚΤ3 clone, BioLegend; for mouse cells: 145-2C11 clone, BD Pharmingen) in complete RPMI medium (20% FBS, Gibco, Thermo Fisher Scientific) prior to T cell staining.

For human Foxp3 evaluation, nuclear staining was performed using the Foxp3 staining buffer set (Miltenyi Biotec) according to the manufacturer’s instructions.

For cytokine production assessment, cells were stimulated with 50 ng/mL PMA (MilliporeSigma), 1 μg/mL ionomycin (MilliporeSigma), and 1 μg/mL GolgiPlug (BD Pharmingen) in complete culture medium for 4 additional hours. For cytokine-producing Th cell evaluation, cells were fixed with PBS 2% paraformaldehyde for 10 minutes at room temperature and intracellularly stained with conjugated antibodies in PBS with 0.05% BSA, 0.01% EDTA, and 0.5% saponin.

The following monoclonal antibodies were used for staining of different markers in human samples: anti-CD14 (clone: M5E2, BioLegend); anti-CD16 (clone: DJ130, Dako); anti-CD25 (clone: 2A3, BD Biosciences); anti-CD4 (clone: SK3, BD Biosciences); anti-CD45RA (clone: H100, BD Biosciences); anti-CD45RO (clone: UCHL1, BD Biosciences); anti-CD66b (clone: G10F5, BioLegend); anti-CD69 (clone: FN50, BD Biosciences), anti-Foxp3 (clone: 3G3, Miltenyi Biotec); anti–HLA-DR (clone: G46-6,BD Biosciences); anti–IFN-γ (clone: B27, BD Biosciences); anti–IL-17A (clone: SCPL1362, BD Biosciences); and anti–IL-22 (clone: 142928, R&D Systems). For mouse samples, the following antibodies were used: anti-CD11b (clone: M1/70, BD Biosciences); anti-CD3 (clone: 145-2C11, BioLegend); anti-CD4 (clone: RM4-5, BD Biosciences); anti-CD45.2 (clone: 104, BioLegend); anti-CD69 (clone: H1.2F3, BD Biosciences); anti-F4/80 (clone: BM8, BioLegend); anti-Gr1 (clone: RB6-8C5, BD Biosciences); anti–IL-17A (clone: TC11-18H10, BD Pharmingen); anti-Ly6C (clone: AL-21, BD Biosciences); and anti-TCRγδ (clone: GL3, BioLegend).

Cells were analyzed in a LSRFortessa or FACSymphony Flow Cytometer, and the data were processed with FlowJo, version 10.0.4 (TreeStar).

### Adoptive transfer of Tregs.

Naive CD4^+^ T cells were purified from single-cell suspensions of the spleen and mesenteric lymph nodes of Foxp3-mRFP/IL17-eGFP/*Cd69^+/+^* or Foxp3-mRFP/IL17-eGFP/*Cd69^–/–^* reporter mice. Cell suspensions were incubated with biotinylated antibodies against CD44, CD8, MHC class II (I-Ab), CD19, B220, IgM, CD11b, CD11c, and DX5 and subsequently with Streptavidin Microbeads (MACS, Miltenyi Biotec). Naive CD4^+^ T cells were negatively selected in MACS LD columns (Miltenyi Biotec) according to the manufacturer’s instructions. The naive status was confirmed by expression of CD4 and CD62L by flow cytometry (data not shown). Naive CD4^+^ T cells (10^6^ cells/mL) were cocultured for 72 hours with irradiated (30 Gy) antigen-presenting cells in the presence of plate-bound anti-CD3 (2 μg/mL) and soluble anti-CD28 (2 μg/mL) plus recombinant TGF-β1 (10 ng/mL) and IL-2 (2 ng/mL) in RPMI medium (Gibco, Thermo Fisher Scientific). After determining the purity of CD4^+^CD25^+^Foxp3^+^ cells, 1.5 × 10^6^ iTregs were intravenously injected into *Cd69^–/–^* mice 4–6 hours after LAD ligation. In the indicated experimental groups, ARL 67158 (0.26 mg/kg mice, Tocris) was intraperitoneally administered in PBS 4–6 hours, 48 hours, and 96 hours after MI. Mice treated with PBS alone were used as controls. Mice were sacrificed 7 days after infarction.

### Cocultures of Tregs and γδT cells.

Single-cell suspensions of peripheral (axillary, inguinal, and submaxillary) lymph nodes from Foxp3-mRFP/IL17-eGFP/*Cd69^+/+^* or Foxp3-mRFP/IL17-eGFP/*Cd69^–/–^* reporter mice were stained with fluorochrome-conjugated antibodies. Tregs (CD3^+^Foxp3-mRFP^+^) and γδT cells (CD3^+^ TCRγδ^+^ cells) were sorted using a BD FACSAria II cell sorter. Both cell populations were cocultured at different ratios for 24 hours in the presence of plate-bound anti-CD3 (2 μg/mL) and soluble anti-CD28 (2 μg/mL) plus recombinant IL-2 (10 ng/mL) with or without ARL 67158 (250 μM, Tocris). Apoptosis of γδT cells was evaluated by flow cytometry using annexin V (BD Biosciences). For IL-17A–eGFP production assessment, cells were incubated for 4 additional hours in the presence of PMA (MilliporeSigma), ionomycin (MilliporeSigma), and GolgiPlug (BD Pharmingen).

### Extracellular ATP consumption assay.

Sorted Tregs (CD4^+^Foxp3-mRFP^+^; 5 × 10^4^ cells) from spleens were activated for 3 days in the presence of plate-bound anti-CD3 (3 μg/mL) and soluble anti-CD28 (2 μg/mL) plus recombinant IL-2 (10 ng/mL) in 96-well U-fond plates. After washing with PBS, cells were incubated for 40 minutes at 37°C in complete RPMI medium (20% FBS, Gibco, Thermo Fisher Scientific) in the presence of ATP (50 μM) and, when indicated, ARL 67158 (250 μM, Tocris). Supernatants (50 μL) were collected at 20 and 40 minutes for extracellular ATP measurements. Cells were washed and used for FACS or qPCR analyses.

The ATP concentration was quantified from the supernatants by firefly luciferase assay in a 96-well luminescence reading plate (Costar). Cell supernatant (50 μL) was diluted in 130 μL Buffer A (150 mM KCl, 25 mM Tris HCl, 1 mM EDTA, 0.1 % BSA fatty acid free, 10 mM KH_2_PO_4_, pH 7.4). The reaction was started by adding 20 μL luciferin/luciferase cocktail (0.5 M tris-acetate, 0.8 mM luciferin, 10 μg/mL luciferase, pH 7.4). Bioluminescent signal was read by Orion Microplate Luminometer with Simplicity 4.2 software. A regression curve for the ATP concentration was calculated by a standard ATP curve measured in parallel in the same assay buffer.

### RNA extraction and qPCR.

RNA was extracted from frozen human PBLs with a MiRNeasy Mini Kit (QIAGEN) as recommended by the manufacturer. Reverse transcription was performed with 200 ng DNAse-treated RNA using the High Capacity cDNA RT Kit (Applied Biosystems). Then, gene expression was measured by real-time qPCR using SYBR Green PCR Mix (Applied Biosystems) and mRNA-specific primers (Thermo Fisher Scientific) for *Cd69* (forward: 5′-ATTGTCCAGGCCAATACACATT-3′, reverse: 5′-CCTCTCTACCTGCGTATCGTTTT-3′); *Foxp3* (forward: 5′-GAGAAGCTGAGTGCCATGCA-3′, reverse: 5′-GGAGCCCTTGTCGGATGAT-3′); *Gapdh* (forward: 5′-GAAGGTGAAGGTCGGAGTC-3′, reverse: 5′-GAAGATGGTGATGGGATTTC-3′); and *Actb* (forward: 5′-CATCGAGCACGGCATCGTCA-3′, reverse: 5′-TAGCACAGCCTGGATAGCAAC-3′). Real-time qPCR analyses were performed with an ABI Prism 7900HT SDS 384-well thermal cycler (Applied Biosystems). Relative gene expression was determined using the 2^–ΔCt^ method and normalized to both *Actb* and *Gapdh* housekeeping genes.

### Statistics.

For statistical analysis, the normality of the distributions was first evaluated using the Shapiro-Wilk test for mouse experiments and the D’Agostino-Pearson test for patient samples with higher *n* values. If normal, an unpaired Student’s *t* test was used for 2-group comparisons or a 1-way ANOVA with Tukey’s post hoc test when more than 2 groups were compared. If distributions were non-normal, a Mann-Whitney’s *U* test was used for the analysis of 2 groups and a Kruskal-Wallis test with Dunn’s post hoc test for multiple comparisons. In the kinetic experiments, a 2-way ANOVA with Šidák’s multiple-comparison post hoc test was performed. Spearman′s correlation coefficient was used to analyze the independence of continuous variables. Associations between nominal variables were calculated by χ^2^ test. Data analyses were performed with GraphPad Prism 8.0 (GraphPad Software). To assess the association of CD69^+^ Tregs with the development of HF, multivariate logistic regression analysis was performed, adjusting for sex, age, troponin levels (normalized values), CK, and ejection fraction with R statistical software, version 3.6.2. In general, *P* values below 0.05 were considered significant.

### Study approval.

Verbal informed consent was obtained from patients who required emergency coronary angiography and primary angioplasty, and written informed consent was obtained immediately after the procedure. All participants included in the study were identified by number and provided authorization for the use of their medical records for research. The study was approved by the research ethics committees of the Universitario de la Princesa and Santa Creu i Sant Pau hospitals. All animal procedures were approved by the ethics committee of the Comunidad Autónoma de Madrid and conducted in accordance with the institutional guidelines that comply with European Institutes of Health directives (European Institutes of Health, 2010).

## Author contributions

PM and RBD contributed to the study design, data analysis, and manuscript preparation. RBD, LMA, and A Curtabbi executed and analyzed preclinical experiments. RBD, IRA, RSD, RJA, and LMA contributed to patient blood sample processing and FACS analysis. RBD analyzed the human data and coordinated the clinical database. MVO, JAE, SCR and FSM provided scientific guidance. HDLF, CR, MMGG, AV, FR, JC, LJJB, A Cecconi, ADC, MT, JA, HB, FA, and JMG provided clinical guidance and/or contributed to patients’ sample collection and data analysis. PM contributed to study conception. All authors read and approved the final manuscript.

## Supplementary Material

Supplemental data

## Figures and Tables

**Table 1:**
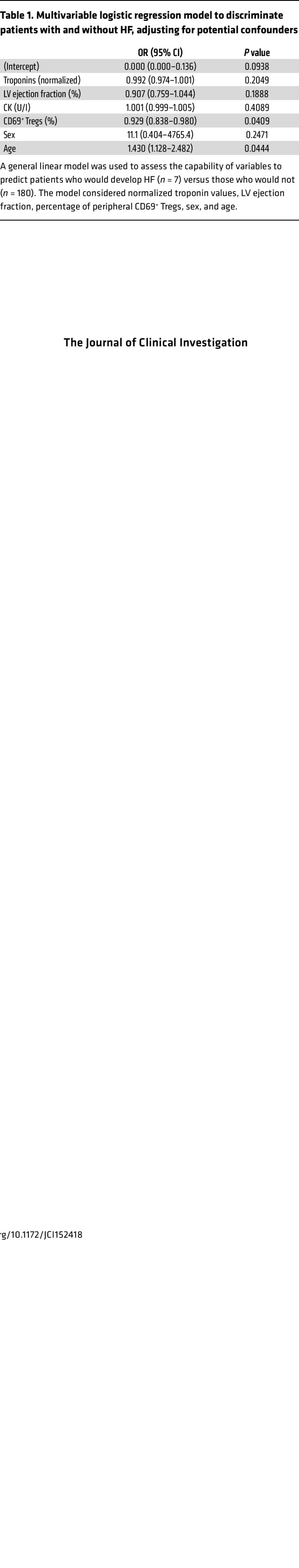
Multivariable logistic regression model to discriminate patients with and without HF, adjusting for potential confounders

## Version 1 09/06/2022

In-Press Preview

## Version 2 11/01/2022

Electronic publication

## References

[1] Swirski FK, Nahrendorf M (2013). Leukocyte behavior in atherosclerosis, myocardial infarction, and heart failure. Science.

[2] Swirski FK, Nahrendorf M (2018). Cardioimmunology: the immune system in cardiac homeostasis and disease. Nat Rev Immunol.

[3] Hofmann U (2012). Activation of CD4^+^ T lymphocytes improves wound healing and survival after experimental myocardial infarction in mice. Circulation.

[4] Hofmann U, Frantz S (2016). Role of T-cells in myocardial infarction. Eur Heart J.

[5] Weiß E (2022). Myocardial-Treg crosstalk: how to tame a wolf. Front Immunol.

[6] Rieckmann M (2019). Myocardial infarction triggers cardioprotective antigen-specific T helper cell responses. J Clin Invest.

[7] Wang Y (2019). C-X-C motif chemokine receptor 4 blockade promotes tissue repair after myocardial infarction by enhancing regulatory t cell mobilization and immune-regulatory function. Circulation.

[8] Seropian IM (2013). Galectin-1 controls cardiac inflammation and ventricular remodeling during acute myocardial infarction. Am J Pathol.

[9] Weirather J (2014). Foxp3^+^ CD4^+^ T cells improve healing after myocardial infarction by modulating monocyte/macrophage differentiation. Circ Res.

[10] Xia N (2015). Activated regulatory T-cells attenuate myocardial ischaemia/reperfusion injury through a CD39-dependent mechanism. Clin Sci (Lond).

[11] George J (2012). Regulatory T cells and IL-10 levels are reduced in patients with vulnerable coronary plaques. Atherosclerosis.

[12] Sardella G (2007). Frequency of naturally-occurring regulatory T cells is reduced in patients with ST-segment elevation myocardial infarction. Thromb Res.

[13] Mao X (2019). IL-37 plays a beneficial role in patients with acute coronary syndrome. Mediators Inflamm.

[14] Klingenberg R (2015). Clonal restriction and predominance of regulatory T cells in coronary thrombi of patients with acute coronary syndromes. Eur Heart J.

[15] Cortes JR (2014). Maintenance of immune tolerance by Foxp3+ regulatory T cells requires CD69 expression. J Autoimmun.

[16] Sanchez-Diaz R (2017). Thymus-derived regulatory T cell development is regulated by C-type lectin-mediated BIC/microRNA 155 expression. Mol Cell Biol.

[17] Martin P, Sanchez-Madrid F (2011). CD69: an unexpected regulator of TH17 cell-driven inflammatory responses. Sci Signal.

[18] Tsilingiri K (2019). Oxidized low-density lipoprotein receptor in lymphocytes prevents atherosclerosis and predicts subclinical disease. Circulation.

[19] Cruz-Adalia A (2010). CD69 limits the severity of cardiomyopathy after autoimmune myocarditis. Circulation.

[20] Barry SP (2013). Enhanced IL-17 signalling following myocardial ischaemia/reperfusion injury. Int J Cardiol.

[21] Mora-Ruiz MD (2019). Role of interleukin-17 in acute myocardial infarction. Mol Immunol.

[22] Yan X (2013). Temporal dynamics of cardiac immune cell accumulation following acute myocardial infarction. J Mol Cell Cardiol.

[23] Yan X (2012). Deleterious effect of the IL-23/IL-17A axis and γδT cells on left ventricular remodeling after myocardial infarction. J Am Heart Assoc.

[24] Chen XM (2018). Gene expression pattern of TCR repertoire and alteration expression of IL-17A gene of γδ T cells in patients with acute myocardial infarction. J Transl Med.

[25] de la Fuente H (2014). The leukocyte activation receptor CD69 controls T cell differentiation through its interaction with galectin-1. Mol Cell Biol.

[26] Lin CR (2015). Glycosylation-dependent interaction between CD69 and S100A8/S100A9 complex is required for regulatory T-cell differentiation. FASEB J.

[27] Sreejit G (2020). Neutrophil-derived S100A8/A9 amplify granulopoiesis after myocardial infarction. Circulation.

[28] Hosono M (2003). Increased expression of T cell activation markers (CD25, CD26, CD40L and CD69) in atherectomy specimens of patients with unstable angina and acute myocardial infarction. Atherosclerosis.

[29] Pasqui AL (2003). T cell activation and enhanced apoptosis in non-ST elevation myocardial infarction. Clin Exp Med.

[30] Ibanez B (2018). 2017 ESC Guidelines for the management of acute myocardial infarction in patients presenting with ST-segment elevation: The Task Force for the management of acute myocardial infarction in patients presenting with ST-segment elevation of the European Society of Cardiology (ESC). Eur Heart J.

[31] Shiow LR (2006). CD69 acts downstream of interferon-alpha/beta to inhibit S1P1 and lymphocyte egress from lymphoid organs. Nature.

[32] Bankovich AJ (2010). CD69 suppresses sphingosine 1-phosophate receptor-1 (S1P1) function through interaction with membrane helix 4. J Biol Chem.

[33] Bohl S (2009). Refined approach for quantification of in vivo ischemia-reperfusion injury in the mouse heart. Am J Physiol Heart Circ Physiol.

[34] Stieger P (2017). Targeting of extracellular RNA reduces edema formation and infarct size and improves survival after myocardial infarction in mice. J Am Heart Assoc.

[35] Sano S (2018). Tet2-mediated clonal hematopoiesis accelerates heart failure through a mechanism involving the IL-1β/NLRP3 inflammasome. J Am Coll Cardiol.

[36] Stevenson MD (2019). NADPH oxidase 4 regulates inflammation in ischemic heart failure: role of soluble epoxide hydrolase. Antioxid Redox Signal.

[37] Bansal SS (2019). Dysfunctional and proinflammatory regulatory T-lymphocytes are essential for adverse cardiac remodeling in ischemic cardiomyopathy. Circulation.

[38] Gonzalez-Amaro R (2013). Is CD69 an effective brake to control inflammatory diseases?. Trends Mol Med.

[39] Papotto PH (2017). IL-17^+^ γδ T cells as kick-starters of inflammation. Nat Immunol.

[40] Huber SA (2000). T cells expressing the gamma delta T cell receptor induce apoptosis in cardiac myocytes. Cardiovasc Res.

[41] Swirski FK (2009). Identification of splenic reservoir monocytes and their deployment to inflammatory sites. Science.

[42] Park SG (2010). T regulatory cells maintain intestinal homeostasis by suppressing γδ T cells. Immunity.

[43] Kunzmann V (2009). Inhibition of phosphoantigen-mediated gammadelta T-cell proliferation by CD4^+^ CD25^+^ FoxP3^+^ regulatory T cells. Immunology.

[44] Borsellino G (2007). Expression of ectonucleotidase CD39 by Foxp3^+^ Tregs: hydrolysis of extracellular ATP and immune suppression. Blood.

[45] Deaglio S (2007). Adenosine generation catalyzed by CD39 and CD73 expressed on regulatory T cells mediates immune suppression. J Exp Med.

[46] Fabbiano S (2015). Immunosuppression-independent role of regulatory T cells against hypertension-driven renal dysfunctions. Mol Cell Biol.

[47] Wang YM (2012). Regulatory T cells participate in CD39-mediated protection from renal injury. Eur J Immunol.

[48] Mascanfroni ID (2015). Metabolic control of type 1 regulatory T cell differentiation by AHR and HIF1-α. Nat Med.

[49] Cibrian D (2016). CD69 controls the uptake of L-tryptophan through LAT1-CD98 and AhR-dependent secretion of IL-22 in psoriasis. Nat Immunol.

[50] Sharir R (2014). Experimental myocardial infarction induces altered regulatory T cell hemostasis, and adoptive transfer attenuates subsequent remodeling. PLoS One.

[51] Pfeffer MA (1993). Development and prevention of congestive heart failure following myocardial infarction. Circulation.

[52] Josefowicz SZ (2012). Regulatory T cells: mechanisms of differentiation and function. Annu Rev Immunol.

[53] Shevach EM (2009). Mechanisms of foxp3^+^ T regulatory cell-mediated suppression. Immunity.

[54] Ohta A, Sitkovsky M (2014). Extracellular adenosine-mediated modulation of regulatory T cells. Front Immunol.

[55] Cai M (2011). Transgenic over expression of ectonucleotide triphosphate diphosphohydrolase-1 protects against murine myocardial ischemic injury. J Mol Cell Cardiol.

[56] Wheeler DG (2012). Transgenic swine: expression of human CD39 protects against myocardial injury. J Mol Cell Cardiol.

[57] Smith SB (2017). Impact of cardiac-specific expression of CD39 on myocardial infarct size in mice. Life Sci.

[58] Kohler D (2007). CD39/ectonucleoside triphosphate diphosphohydrolase 1 provides myocardial protection during cardiac ischemia/reperfusion injury. Circulation.

[59] Lang RM (2015). Recommendations for cardiac chamber quantification by echocardiography in adults: an update from the American Society of Echocardiography and the European Association of Cardiovascular Imaging. Eur Heart J Cardiovasc Imaging.

