# Supplementary material for: CD69 expression on regulatory T cells protects from immune damage after myocardial infarction
Source: J Clin Invest. 2022 Nov 1;132(21):e152418. doi: 10.1172/JCI152418 (PMC9621142; doi:10.1172/JCI152418)
Supplement: Supplemental data [file jci-132-152418-s033.pdf]

SUPPLEMENTAL MATERIAL

CD69 expression on regulatory T cells protects from immune damage after myocardial infarction

Single sentence summary: CD69$^+$ Treg cells protect from myocardial damage

Rafael Blanco-Domínguez [1], Hortensia de la Fuente [2,3], Cristina Rodríguez [3,4,5], Laura Martín-Aguado [1], Raquel Sánchez-Díaz [1, 3], Rosa Jiménez-Alejandre [1], Iker Rodríguez-Arabaolaza [1], Andrea Curtabbi[1], Marcos M. García-Guimaraes [6], Alberto Vera [6], Fernando Rivero [6], Javier Cuesta [6], Luis J. Jiménez-Borreguero [3,6], Alberto Cecconi [6], Albert Duran [7], Manel Taurón [7], Judith Alonso [3, 5, 8], Héctor Bueno [1, 3, 9, 10], María Villalba-Orero [1, 11], Jose Antonio Enríquez [1, 12], Simon C Robson [13], Fernando Alfonso [3, 6], Francisco Sánchez-Madrid [1, 2, 3], José Martínez-González [3, 5, 8] and Pilar Martín [1, 3,*]

[1] *Vascular Pathophysiology Area, Centro Nacional de Investigaciones Cardiovasculares (CNIC),* Madrid, Spain.

[2] *Department of Immunology, IIS Princesa, Hospital Universitario de la Princesa, Universidad Autónoma de Madrid,* Madrid, Spain.

[3] *CIBER de Enfermedades Cardiovasculares (CIBERCV),* Madrid, Spain.

[4] *Institut de Recerca del Hospital de la Santa Creu i Sant Pau,* Barcelona, Spain.

[5] *Instituto de Investigación Biomédica Sant Pau (IIB-Sant Pau),* Barcelona, Spain

[6] *Department of Cardiology, IIS Princesa, Hospital Universitario de la Princesa, Universidad Autónoma de Madrid,* Madrid, Spain.

[7] *Hospital de la Santa Creu i Sant Pau,* Barcelona, Spain.

[8] *Instituto de Investigaciones Biomédicas de Barcelona -Consejo Superior de Investigaciones Científicas (IIBB-CSIC),* Barcelona, Spain.

[9] *Cardiology Department, Hospital Universitario 12 de Octubre and Instituto de Investigación Sanitaria Hospital 12 de Octubre (imas12),* Madrid, Spain.

[10] *Facultad de Medicina, Universidad Complutense de Madrid, Madrid, Spain.*

[11]*Departamento de Medicina y Cirugía Animal, Facultad de Veterinaria, Universidad Complutense de Madrid;* Madrid, Spain.

[12] *CIBER de Fragilidad y Envejecimiento Saludable (CIBERFES),* Madrid, Spain.

[13] *Department of Medicine, Harvard Medical School, Transplantation Research Center, Beth Israel Deaconess Medical Center, Boston, MA, USA.*

Address for Correspondence:

Pilar Martín, PhD

Vascular Pathophysiology Area, Centro Nacional de Investigaciones Cardiovasculares (CNIC). Calle Melchor Fernández Almagro, 3. 28029 Madrid, Spain.

Tel: +34 914531200

e-mail: pmartinf@cnic.es.

Supplemental Figures

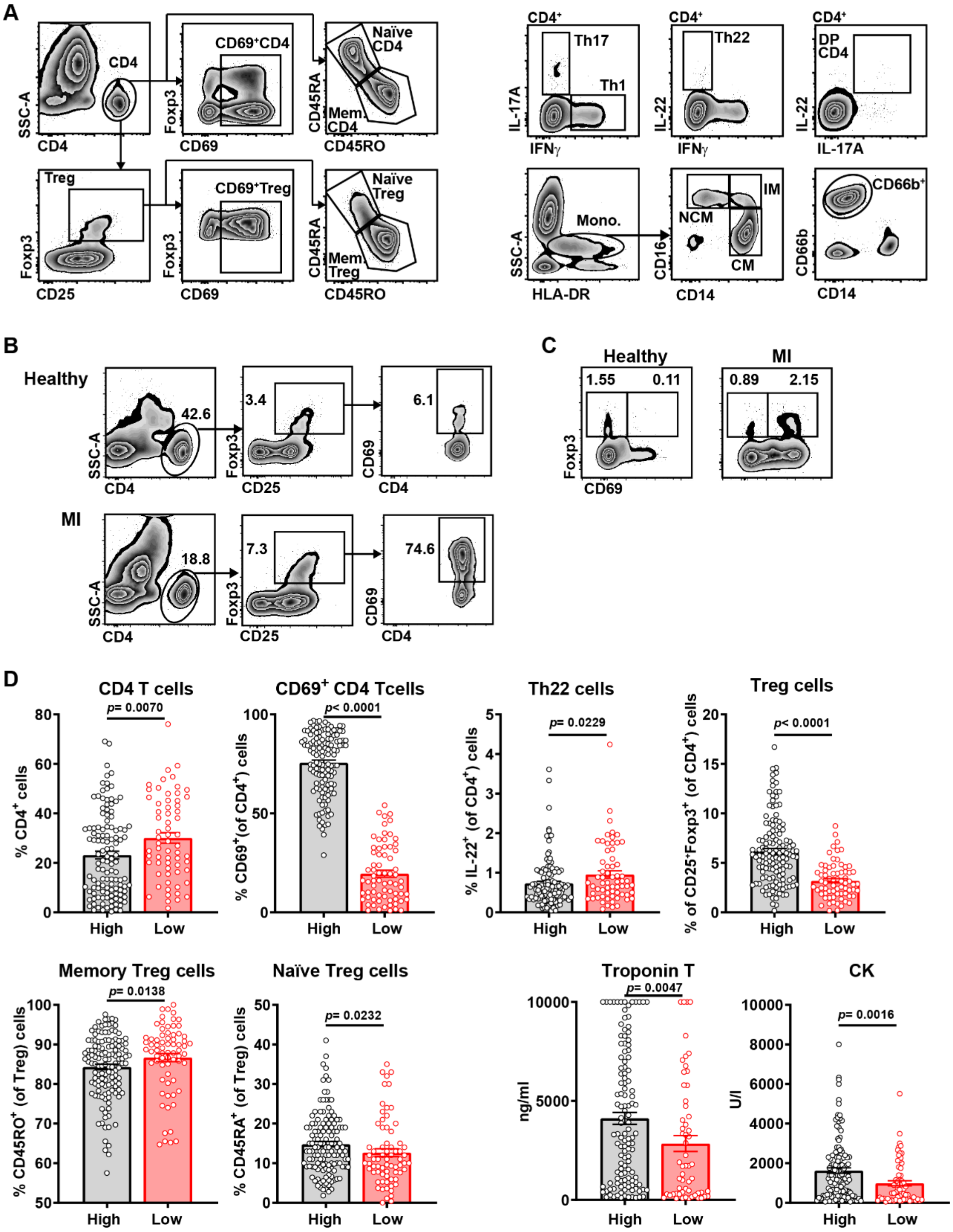

Supplemental Figure 1. **FACS analysis of human peripheral blood leukocytes.** (**A**) Gating strategy for immune cell population analysis, after excluding doublets and autofluorescent cells.

DP, IL-17A$^+$IL-22$^+$ Double Positive CD4$^+$ cells; Mem., Memory; Mono., Monocytes; CM, Classical Monocytes; NCM, Non-classical Monocytes; IM, Intermediate Monocytes. (**B**) Representative zebra plot of CD4$^+$ T cells, after excluding doublets and autofluorescent cells. After gating on CD4$^+$ cells, Treg cells were determined as double positive for Foxp3 and CD25 markers. (**C**) Representative zebra plot of Foxp3$^+$CD69$^-$ and Foxp3$^+$CD69$^+$ cells out of total cells. Percentages appear inbox. (**D**) Quantification of different immune populations, measured by FACS, and cardiac damage markers in MI patients with >50% of CD69$^+$ Treg cells (High) and MI patients with <50% of CD69$^+$ Treg cells (Low). Individual data points and mean ±SEM are represented for each group of patients. Data were analyzed by Mann-Whitney U-test.

A

	Basal			Isoproterenol		
	Cd69$^{+/+}$	*Cd69*$^{-/-}$	*P*-value	*Cd69*$^{+/+}$	*Cd69*$^{-/-}$	*P*-value
N	6	8		6	8	
Arrhythmias, %	33.3	37.5	0.8721	33.3	87.5	**0.0363**
Ventricular premature depolarization (VPD), %	33.3	37.5	0.8721	33.3	50.0	0.5329
>10 VPD during acquisition, %	0.0	25.0	0.1859	16.7	25.0	0.7069
First degree of atrioventricular block, %	0.0	0.0	-	66.7	87.5	0.3472
Sionatrial pauses, %	16.7	0.0	0.2308	0.0	50.0	**0.0404**
ST-segment elevation, %	16.7	12.5	0.8255	0.0	50.0	**0.0404**
Death during evaluation, %	0.0	0.0	-	0.0	50.0	**0.0404**

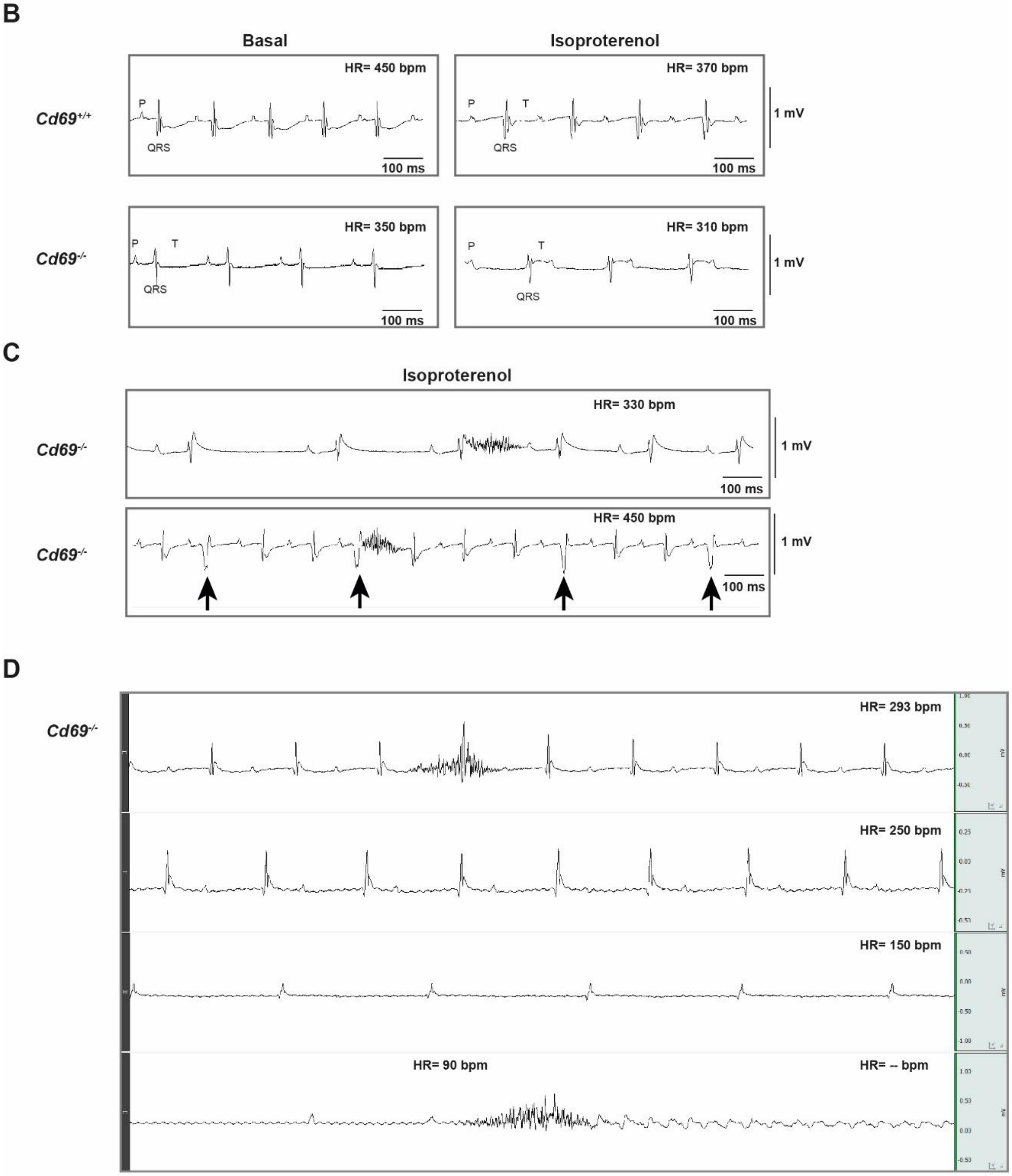

Supplemental Figure 2. Isoproterenol-induced ST elevation and abnormal conduction in *Cd69*$^{-/-}$ mice. (**A**) Surface electrocardiograms were recorded in anesthetized *Cd69*$^{+/+}$ and *Cd69*$^{-/-}$ mice before isoproterenol (basal) and after isoproterenol injection two days after LAD-ligation. The table summarizes the main electrocardiogram alterations. Four mice out of eight mice died shortly after the isoproterenol. (**B**) Representative electrocardiograms showing ST elevation in *Cd69*$^{-/-}$ mice when isoproterenol was injected. (**C**) After isoproterenol injection, abnormal sinoatrial conduction and ventricular beats (arrows) were frequently observed in *Cd69*$^{-/-}$ mice. (**D**) Representative electrocardiogram over time from an infarcted mouse that died during acquisition and after isoproterenol administration. The total interval of time from isoproterenol administration and death was 20-25 minutes.

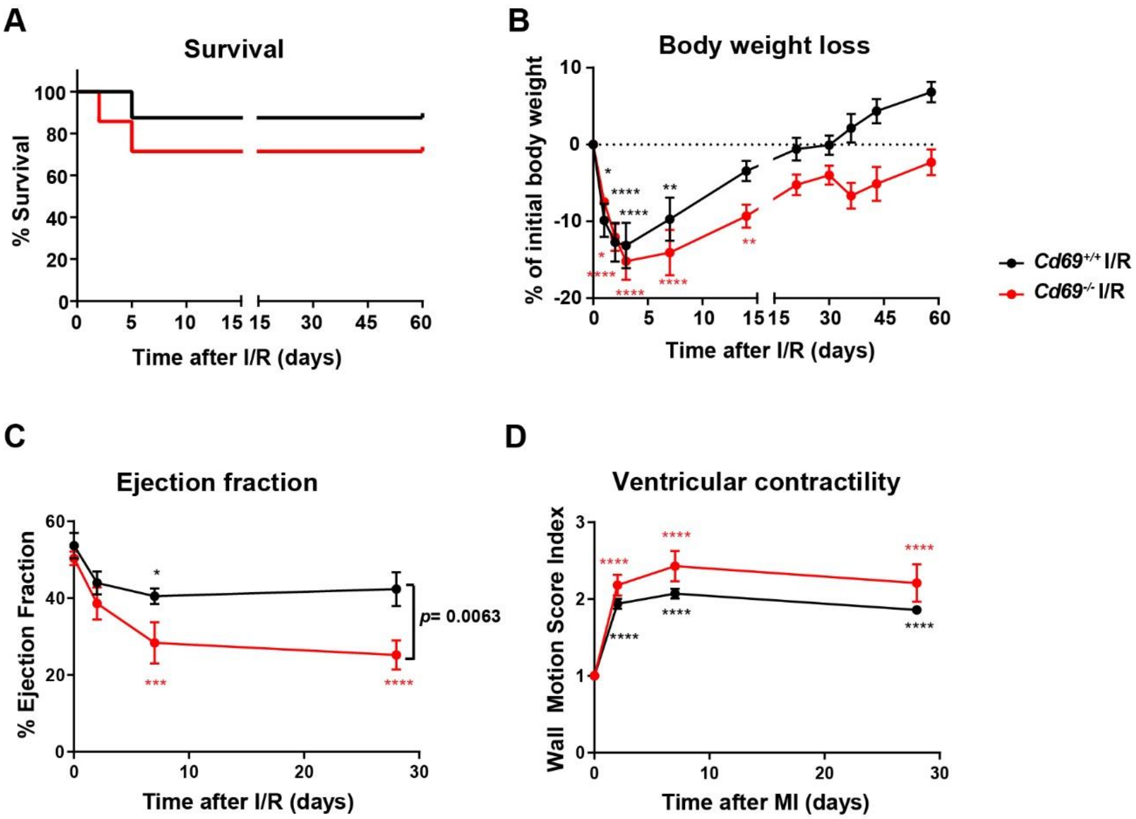

Supplemental Figure 3. CD69 deficiency worsens left ventricular systolic dysfunction and progression after myocardial ischemia/reperfusion injury in mice. (**A**) Survival curve of mice after LAD-ligation (n= 11). (**B**) Kinetics of the percentage of body weight loss after LAD-ligation (n= 11). (**C-D**) Time course of the left ventricular dysfunction as a function of the (**C**) left ventricular ejection fraction and (**D**) the Wall Motion Score Index measured by echocardiography (n= 11). Data in **B-D** represent means ± SEM and were analyzed by two-way ANOVA with Sidak's multiple comparisons test. Differences between each day and day 0 are indicated with asterisks ($p < 0.05$, **$p < 0.01$, ***$p < 0.001$, ****$p < 0.0001$), in black for *Cd69*$^{+/+}$ and in red for *Cd69*$^{-/-}$. Data were pooled from two independent experiments.

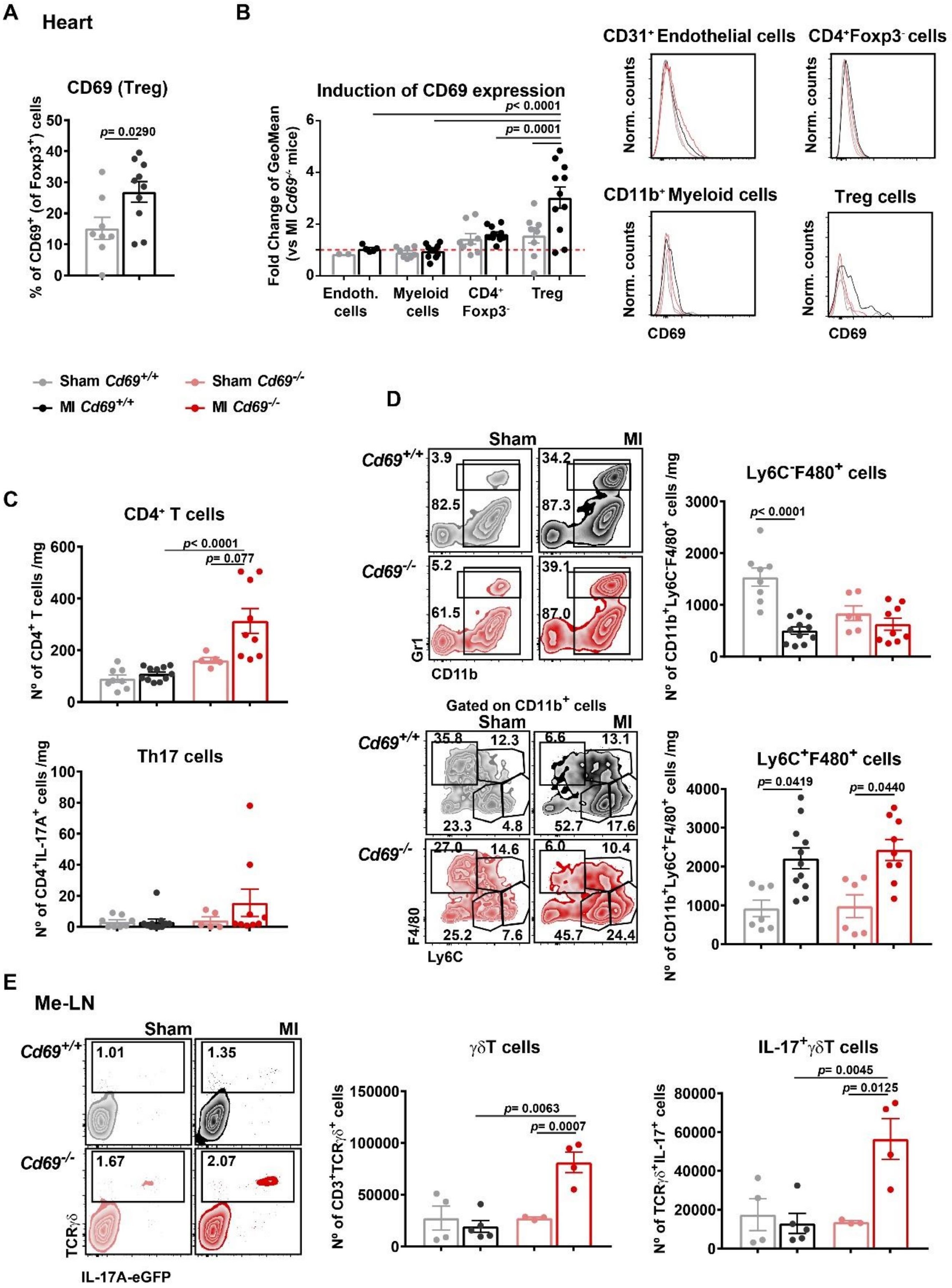
A
Heart
CD69 (Treg)
p= 0.0290
% of CD69+ (of Foxp3+) cells
B
Induction of CD69 expression
p< 0.0001
p= 0.0001
Fold Change of GeoMean (vs MI Cd69-/- mice)
Endoth. cells
Myeloid cells
CD4+ Foxp3-
Treg
CD31+ Endothelial cells
CD4+Foxp3- cells
CD11b+ Myeloid cells
Treg cells
Norm. counts
CD69
Sham Cd69+/+
Sham Cd69-/-
MI Cd69+/+
MI Cd69-/-
C
CD4+ T cells
p< 0.0001
p= 0.077
Nº of CD4+ T cells /mg
Th17 cells
Nº of CD4+IL-17A+ cells /mg
D
Sham
MI
Cd69+/+
Cd69-/-
Gr1
CD11b
Ly6C-F480+ cells
p< 0.0001
Nº of CD11b+Ly6C-F4/80+ cells /mg
Gated on CD11b+ cells
F4/80
Ly6C
Ly6C+F480+ cells
p= 0.0419
p= 0.0440
Nº of CD11b+Ly6C+F4/80+ cells /mg
E
Me-LN
TCRγδ
IL-17A-eGFP
γδT cells
p= 0.0063
p= 0.0007
Nº of CD3+TCRγδ+ cells
IL-17+γδT cells
p= 0.0045
p= 0.0125
Nº of TCRγδ+IL-17+ cells

Supplemental Figure 4. Heart infiltrating cells and γδT cells in the mediastinal lymph nodes after myocardial infarction. (**A**) Quantification of the percentages of $CD69^+$ cells in the heart, after gating on $CD4^+Foxp3^+$ Treg cells (n= 8-10). (**B**) Histograms show the representative expression of CD69 on $CD45^-CD31^+$ endothelial cells, $CD45^+CD11b^+$ myeloid cells, $CD45^+CD11b^-CD4^+Foxp3^-$ T effector cells and $CD45^+CD11b^-CD4^+Foxp3^+$ Treg cells in the heart two days after MI. Quantification of the fold change of CD69 geometric mean (GeoMean) of expression in the populations above. Each population was normalized versus the GeoMean of Cd69-/- mice with MI (red line), as considered the fluorescence baseline. Data are represented as means +- SEM and were analyzed by two-way ANOVA with Sidak's multiple comparisons test (n= 8-11). (**C**) Numbers of $CD4^+$ T cells and Th17 cells per mg of tissue in the heart (n= 5-11). (**D**) Gating strategy with representative percentages of heart infiltrating myeloid populations (right, after gating on $CD45^+$ cells; left, after gating on $CD45^+CD11b^+$ cells), quantification of number of these cell populations per mg of tissue are shown below (n= 6-11). (**E**) Representative density plots and absolute numbers of γδT cells and $Il\text{-}17^+$ γδT cells in the mediastinal lymph nodes (Me-LN) (n= 4-5). Representative graphs of three independent experiments that were performed two days post LAD-ligation/sham surgery. Data are represented as means ± SEM and were analyzed by one-way ANOVA with Tukey's multiple comparisons test or, for non-normal distributions, by Kruskal-Wallis with Dunn's multiple comparisons test.

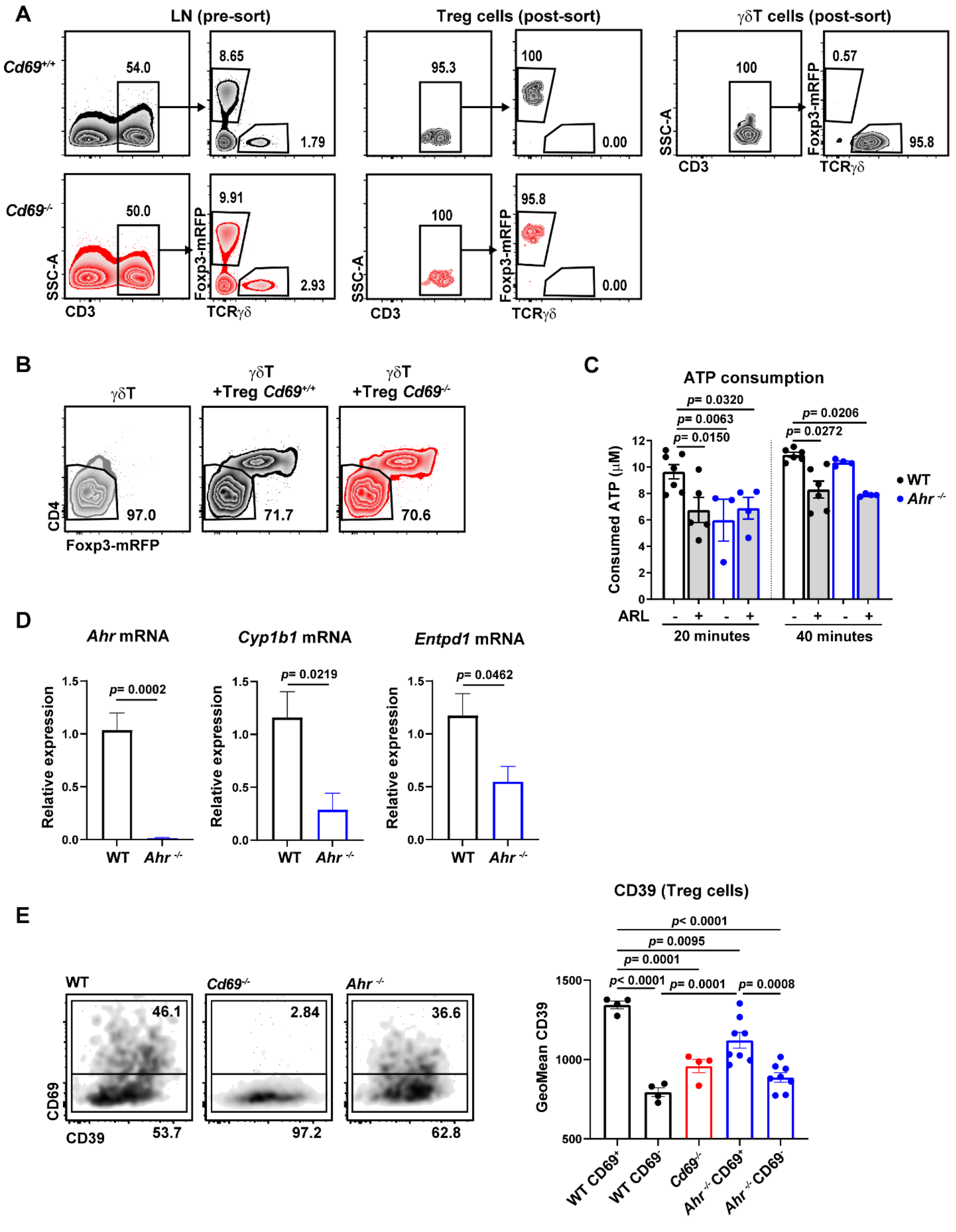
A
LN (pre-sort)
Treg cells (post-sort)
γδT cells (post-sort)
Cd69+/+
Cd69-/-
54.0
8.65
1.79
50.0
9.91
2.93
95.3
100
0.00
100
95.8
0.00
100
0.57
95.8
SSC-A
CD3
Foxp3-mRFP
TCRγδ
B
γδT
γδT +Treg Cd69+/+
γδT +Treg Cd69-/-
97.0
71.7
70.6
CD4
Foxp3-mRFP
C
ATP consumption
p= 0.0320
p= 0.0063
p= 0.0150
p= 0.0206
p= 0.0272
Consumed ATP (μM)
WT
Ahr -/-
ARL
20 minutes
40 minutes
D
Ahr mRNA
Cyp1b1 mRNA
Entpd1 mRNA
p= 0.0002
p= 0.0219
p= 0.0462
Relative expression
WT
Ahr -/-
E
WT
Cd69-/-
Ahr -/-
46.1
53.7
2.84
97.2
36.6
62.8
CD69
CD39
CD39 (Treg cells)
p< 0.0001
p= 0.0095
p= 0.0001
p< 0.0001
p= 0.0001
p= 0.0008
GeoMean CD39
WT CD69+
WT CD69-
Cd69-/-
Ahr-/- CD69+
Ahr-/- CD69-

Supplemental Figure 5. **Sorting strategy of γδT and Treg cells and impaired CD39 expression in AhR-deficient Treg cells.** (**A**) Sorting strategy, representative frequencies in peripheral lymph nodes (LN) and purity of sorted γδT cells and Treg cells for co-culture experiments. (**B**) After 24 hours co-culture, apoptosis and IL-17A production was assessed on the γδT cell population as indicated in the density plots, ratios γδT:Treg of 1:0 (γδT cells alone) and 1:0.5 is shown. (**C**) Extracellular ATP was measured in the supernatant of isolated and 72h-activated wild-type or *AhR*$^{-/-}$ Treg cells, in the presence or absence of the CD39 inhibitor ARL 67156 (ARL) at the indicated time points after ATP supplementation to the medium (n= 4-7). One representative out of three independent experiments. Means ± SEM are shown and data were analyzed by mixed-effects two-way ANOVA with Sidak's multiple comparisons test. (**D**) Quantification of mRNA levels of *Ahr*, *Cyp1b1* and *Entpd1* by qPCR in sorted and activated Treg cells after 40 minutes of incubation with ATP (n= 6-7). Data pooled from two independent experiments are represented as means ± SEM and were analyzed by unpaired t-test. (**E**) Surface CD39 expression on CD69^{+} and CD69^{-} Treg cells from wild-type and *AhR*$^{-/-}$ Treg cells and in *Cd69*$^{-/-}$ Treg cells, measured by FACS after 40 minutes of incubation. Representative density plots and percentages of CD69^{+} and CD69^{-} Treg cells are shown on the left. One representative out of two independent experiments (n= 4-8). Data were analyzed by one-way ANOVA with Tukey's multiple comparisons test.

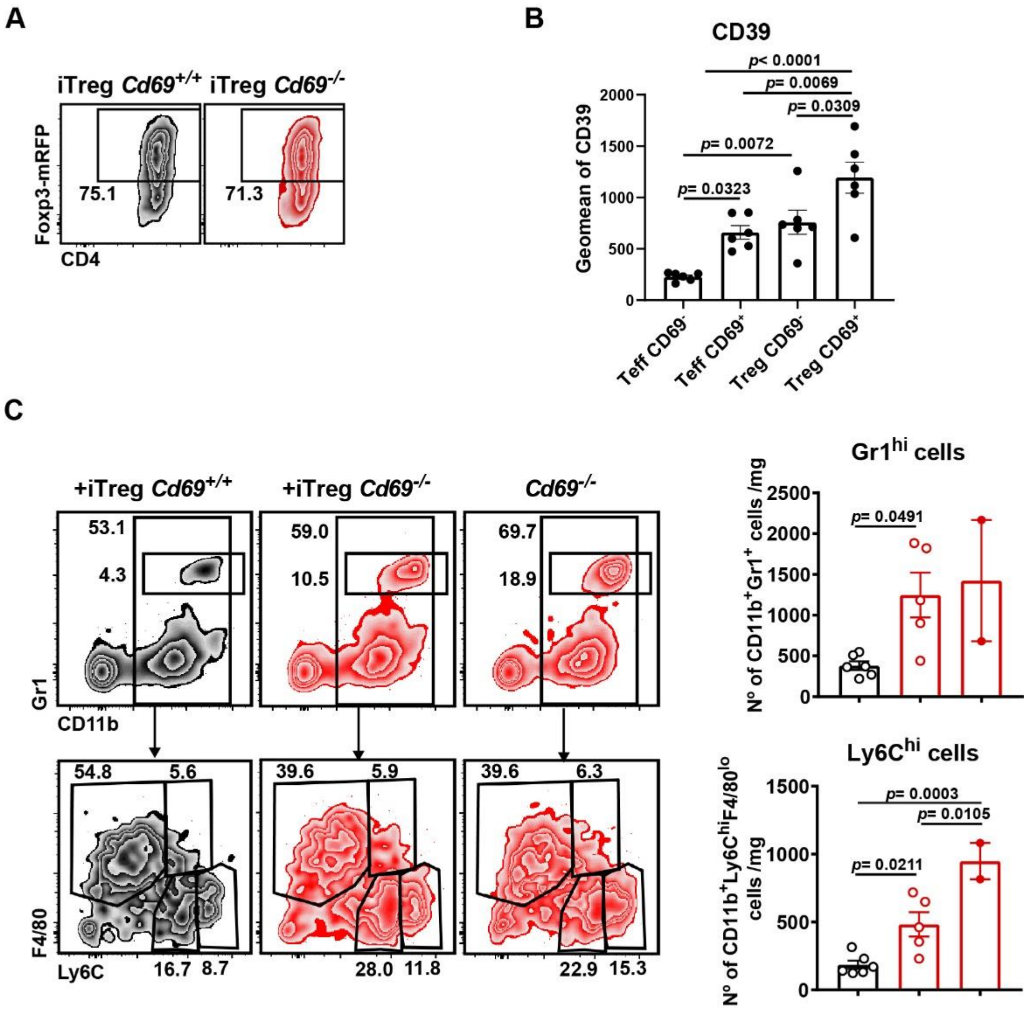

Supplemental Figure 6. **Adoptive transfer of CD69-sufficient Treg cells to CD69-deficient mice.** (**A**). The density plots represent the purity of 72 h-differentiated iTreg cells injected to receptor mice 5-6 h after infarction. (**B**) Quantification of CD39 geomean of expression in $CD69^{+}$or $CD69^{-}$ $CD4^{+}Foxp3^{-}$ T effector (Teff) cells and $CD4^{+}Foxp3^{+}$ regulatory T cells (Treg) in the heart seven days post LAD-ligation and iTreg cell transfer. (n= 6 mice). Data are represented as means ± SEM and were analyzed by one-way ANOVA with Tukey's post hoc test. (**C**) Representative zebra plots and quantification of myeloid cell populaitons in the myocardium 7 days post infarction. Data correspond to one representative out of three independent experiments,

bars indicate means ± SEM and data were analyzed by one-way ANOVA with Tukey's post hoc test.

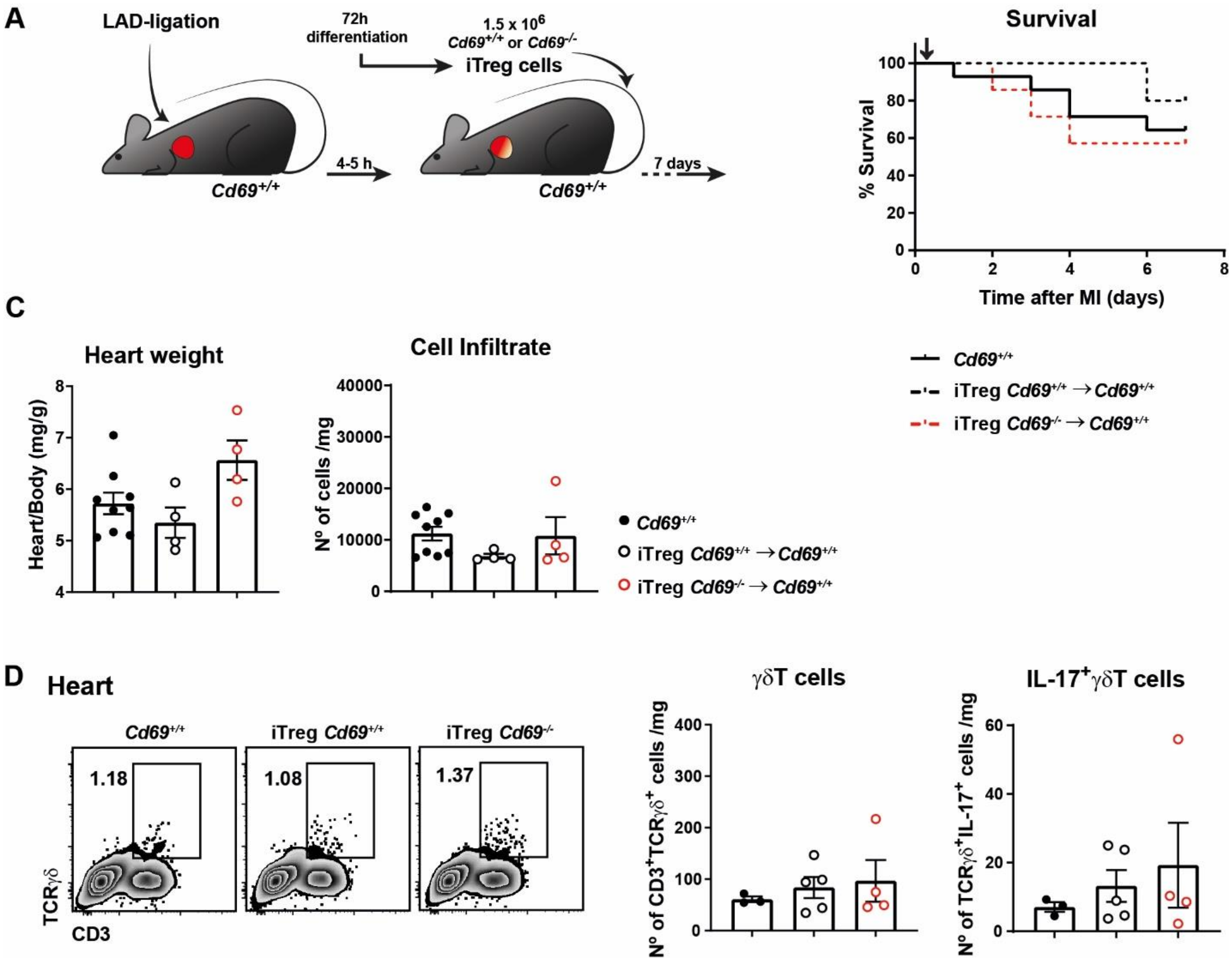

Supplemental Figure 7. **Adoptive transfer of CD69-sufficient Treg cells to wild-type mice.** (**A**) Schematic workflow of the iTreg adoptive transfer after LAD-ligation. (**B**) Survival after LAD-ligation (n= 5-14). Black arrow depicts the time of iTreg cell inoculation (4-5h post-infarction). *Cd69*$^{+/+}$ mice without cell transfer were used as controls. *P*-value was calculated by long-rank (Mantel-Cox) test. (**C**) Heart-to-body weight ratio and total leukocyte number per mg of heart tissue 7 days after LAD-ligation (n= 4-9). (**D**) Representative density plots (gated on CD45^{+}CD11b^{-} cells) and numbers of γδT cells and IL-17^{+} γδT cells per mg of myocardial tissue. Data in **C-D** correspond to one representative out of three independent experiments, bars indicate means ± SEM and data were analyzed by one-way ANOVA with Tukey's post hoc test.

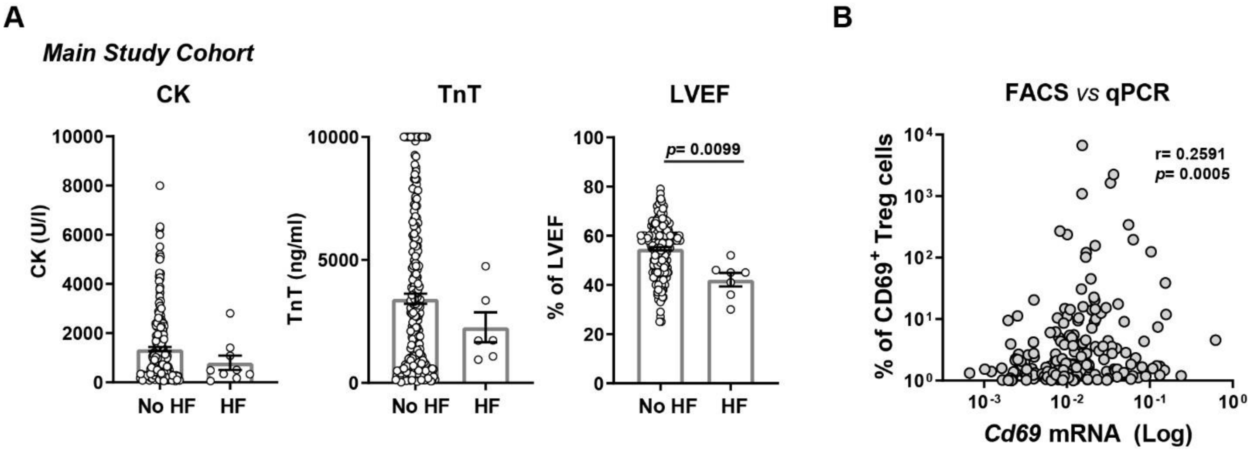

Supplemental Figure 8. Cardiac damage markers in the main study cohort and correlation of qPCR and FACS data for CD69 determination. (**A**) Quantification of creatine kinase (CK), troponin T (TnT) and left-ventricular ejection fraction (LVEF), measured at the time of hospital admission by acute MI, in patients with and without HF from the main study cohort. Data were analyzed by Mann-Whitney U-test. Only p-values < 0.05 are shown. (**B**) Correlation between the percentage of $CD69^+$ Treg cells among peripheral blood leukocytes and the Cd69 mRNA levels in peripheral blood leukocytes, analyzed by Spearman's correlation coefficient (r).

Supplemental Tables

Supplemental Table 1. **Characteristics of the main study population from the Hospital Universitario de La Princesa.** (**A**) Baseline clinical characteristics. (**B**) Clinical presentations and in-hospital evolution. Data are expressed as means ± SD or as number with percentages of patients between brackets. PCI: Percutaneous Coronary Intervention; CABG: Coronary Artery Bypass Grafting; STEMI: ST-elevation Myocardial Infarction; NSTEMI: Non-ST-elevation Myocardial Infarction; RCA: Right Coronary Artery; LAD: Left Anterior Descending Artery; LCX: Left Circumflex Coronary Artery; CRP: C-Reactive Protein.

A. Baseline clinical characteristics

	N= 283 patients
Age (years)	64 ± 13
Sex (male)	212 (74.9)
Ethnicity	
Caucasian	273 (96.5)
Latino American	4 (1.4)
Asian	2 (0.7)
Black	4 (1.4)
Weight (kilograms)	78 ± 16
Height (meters)	168 ± 11
Body Mass Index (kg/m^2)	27 ± 4
Systemic hypertension	153 (54.1)
Hyperlipidemia	163 (57.6)
Diabetes mellitus	63 (22.3)
Smoking habit	
Smoker	123 (43.5)
Ex-smoker	75 (26.5)
Never	86 (30.4)
Family history of ischemic heart disease	21 (7.4)
Peripheral artery disease	15 (5.3)
Chronic kidney disease	22 (7.8)
Ischemic heart disease	34 (12)
Previous myocardial infarction	29 (10.2)

Previous PCI	26 (9.2)
Previous CABG	2 (0.7)
Atrial fibrillation	12 (4.2)

B. Clinical presentation and in-hospital evolution

	N=283 patients
Confirmed final diagnosis	
STEMI	209 (73.9)
NSTEMI	74 (26.1)
Culprit coronary artery	
Left main	8 (2.8)
LAD	114 (40.3)
LCX	43 (15.2)
RCA	115 (40.6)
Bypass	1 (0.4)
Unknown	2 (0.7)
Coronary artery affected	
Left main	12 (4.2)
LAD	165 (58.3)
LCX	98 (34.6)
RCA	166 (58.6)
Number of coronary arteries affected	
1-vessel	174 (61.5)
2-vessels	61 (21.6)
3-vessels	48 (16.9)
Killip-Kimball classification	
I	246 (86.9)
II	21 (7.4)
III	4 (1.4)
IV	12 (4.2)
Revascularization therapy	
PCI	261 (92.2)
CABG	8 (2.8)
Conservative	14 (4.9)
Symptoms to balloon time STEMI (min)	180 (IQR 115-330)
Peak creatine kinase (U/L)	913 (308-1900)
Peak troponin T (ng/ml)	2508 (660-5533)
Left-ventricular ejection fraction (%)	54 ± 10

Reduced left-ventricular ejection fraction (<50%)	88 (31.1)
Acute decompensated Heart Failure	38 (13.4)
New onset of Atrial Fibrillation	12 (4.2)
Fever during admission	33 (11.7)
Antibiotic therapy during admission	33 (11.7)
Peak number of leukocytes (/mm^3)	11820 (9450-14805)
Peak CRP level (mg/L)	2.5 (0.7-11.4)
Peak procalcitonin level (ng/mL)	0.18 (0.08-0.89)

Supplemental Table 2. Correlations between immune populations and cardiac damage biomarkers in MI patients. Spearman correlation coefficient (r) and *P* values were calculated between the percentages or the ratio of percentages of the different immune cell populations, measured by flow cytometry, and the levels of serum troponin T, serum creatine kinase (CK) and left-ventricular (LV) ejection fraction, measured by echocardiography. The number of XY pairs shows the number of samples for each comparison. Significant *P* values are highlighted in bold.

	**Troponin T**			**CK**			**LV Ejection Fraction**		
	r	*P* value	XY pairs	r	*P* value	XY pairs	r	*P* value	XY pairs
**CD4**	**-0.3449**	**<0.0001**	**182**	**-0.3095**	**<0.0001**	**196**	**0.1967**	**0.0062**	**192**
**CD45RA$^+$ CD4**	0.0146	0.8449	182	0.01085	0.88	196	0.0228	0.7527	192
**CD45RO$^+$ CD4**	-0.0146	0.8441	182	-0.0122	0.8646	196	-0.0224	0.7576	192
**CD69$^+$ CD4**	**0.2188**	**0.003**	**182**	**0.1928**	**0.0068**	**196**	-0.1167	0.1068	192
**Treg (CD4$^+$Foxp3$^+$CD25$^+$)**	-9.8E-05	0.999	182	0.0067	0.9254	196	0.0224	0.7568	192
**CD45RA$^+$ Treg**	-0.0129	0.8623	182	0.01409	0.8446	196	-0.0626	0.388	192
**CD45RO$^+$ Treg**	0.0042	0.9547	182	-0.0301	0.6755	196	0.0614	0.397	192
**CD69$^+$ Treg**	**0.1931**	**0.009**	**182**	**0.2042**	**0.0041**	**196**	-0.0659	0.3633	192
**Th1 (CD4$^+$IFNg$^+$)**	-0.1049	0.1814	164	-0.0618	0.4118	178	0.0425	0.5774	174
**Ratio Th1/Treg**	-0.0915	0.2438	164	-0.0542	0.4721	178	0.0049	0.9484	174
**Ratio Th1/CD69$^+$ Treg**	**-0.2117**	**0.0067**	**163**	**-0.1869**	**0.0127**	**177**	0.1113	0.1451	173
**Th17 (CD4$^+$IL-17$^+$)**	-0.0439	0.5775	163	-0.0660	0.3825	177	-0.0094	0.902	173
**Ratio Th17/Foxp3**	-0.0161	0.838	163	-0.0300	0.6913	177	-0.0168	0.8256	173
**Ratio Th17/CD69$^+$ Treg**	**-0.1867**	**0.0167**	**164**	**-0.2398**	**0.0013**	**178**	0.0858	0.2598	174
**CD4$^+$IL17$^+$IL22$^+$ (DP)**	-0.0692	0.378	164	-0.0907	0.2281	178	0.0172	0.8213	174
**Th22 (CD4$^+$IL-22$^+$)**	-0.1104	0.1592	164	-0.1246	0.0975	178	0.1209	0.1121	174
**Ratio Th22/Treg**	-0.0834	0.2852	166	-0.1065	0.1548	180	0.0757	0.318	176
**Ratio Th22/ CD69$^+$ Treg**	**-0.1971**	**0.0107**	**167**	**-0.2354**	**0.0014**	**181**	0.1373	0.0685	177
**Monocytes**	-0.1762	0.1156	81	-0.1942	0.075	85	0.0202	0.8549	84
**Classical Monocytes**	0.2102	0.0613	80	0.05317	0.631	84	-0.1856	0.093	83
**Intermediate Monocytes**	-0.1228	0.2747	81	-0.0681	0.5354	85	0.2214	0.043	84
**Non-classical Monocytes**	-0.2074	0.0632	81	-0.1433	0.1907	85	0.1031	0.3507	84
**CD66b$^+$CD14^{lo}**	0.2158	0.0546	80	0.08271	0.4545	84	-0.0792	0.4761	83

Supplemental Table 3. Follow-up data of patients from the main study cohort from the Hospital Universitario de La Princesa. At medium time of follow-up (2.5 y): 91.9 % free from composite all-cause death or admission for heart failure.

	N=187 patients
Time of follow-up (years)	2.5 (IQR 2.1-3.0)
All-cause death	2 (6.4 %)
New admission for heart failure	7 (3.7 %)

Supplemental Table 4. Characteristics of the validation cohort from the Hospital de la Santa Creu i Sant Pau. Data are expressed as means ± SD or as number with percentages of patients between brackets. (**A**) Baseline clinical characteristics. (**B**) Clinical presentations and in-hospital evolution. Data are expressed as means ± SD or as number with percentages of patients between brackets. STEMI: ST-elevation Myocardial Infarction; NSTEMI: Non-ST-elevation Myocardial Infarction; RCA: Right Coronary Artery; LAD: Left Anterior Descending Coronary Artery; LCX: Left Circumflex Coronary Artery; OM: Obtuse Marginal Coronary artery; PCI: Percutaneous Coronary Intervention.

A. Baseline clinical characteristics.

	N=84 patients
Age (years)	59.6 ±13.6
Sex (male)	64 (76.2)
Ethnicity	
Caucasian	81 (96.42)
Asian	3 (3.57)
Systemic hypertension	44 (52.38)
Hyperlipemia	38 (45.23)
Diabetes mellitus	13 (15.47)
Smoking habit	
Smoker	43 (51.19)
Ex-smoker	16 (19.04)
Never	25 (29.76)
Family history of ischemic heart disease	19 (22.61)*
Peripheral artery disease	6 (7.14)
Chronic kidney disease	4 (4.76)
Ischemic heart disease	8 (9.5)
Previous myocardial infarction	2 (2.4)

*73 patients

B. Clinical presentation and in-hospital evolution.

	N=84 patients
Confirmed diagnosis	
STEMI	82 (97.61)
NSTEMI	2 (2.38)
Culprit coronary artery	

LAD<br>RCA<br>LCX<br>OM<br>Other	40 (47.6)<br>28 (33.3)<br>7 (8.3)<br>6 (7.1)<br>3 (3.6)
Revascularization therapy<br>PCI	 84 (100)
Troponin T (ng/L)	*3605 (204-10000)
Left-Ventricular Ejection Fraction (%)	†48±9.54
Antibiotic therapy during admission	0

*64 patients; †70 patients.

Supplemental Table 5. Follow-up data of patients from the validation cohort from the Hospital de la Santa Creu i Sant Pau.

	N=84 patients
Time of follow-up (years)	2,15 IQR (1,76-2,68)
All-cause death	4 (4.8)
New admission for heart failure	9 (10.71)
